# Vaccines mimicking conformational epitopes on α-synuclein fibrils provide immunity to Parkinson’s disease

**DOI:** 10.1093/brain/awag010

**Published:** 2026-01-09

**Authors:** Liang Ma, Sara Reithofer, Verena Pesch, José Miguel Flores-Fernandez, Aishwarya Sriraman, Caleb Duckering, Sara Amidian, Pelin Özdüzenciler, Laura Müller, Holger Wille, Gültekin Tamgüney

**Affiliations:** Institut für Physikalische Biologie, Mathematisch-Naturwissenschaftliche Fakultät, Heinrich-Heine-Universität Düsseldorf, Düsseldorf 40225, Germany; Institut für Biologische Informationsprozesse, Strukturbiochemie (IBI-7), Forschungszentrum Jülich, Jülich 52425, Germany; Institut für Physikalische Biologie, Mathematisch-Naturwissenschaftliche Fakultät, Heinrich-Heine-Universität Düsseldorf, Düsseldorf 40225, Germany; Institut für Biologische Informationsprozesse, Strukturbiochemie (IBI-7), Forschungszentrum Jülich, Jülich 52425, Germany; Institut für Physikalische Biologie, Mathematisch-Naturwissenschaftliche Fakultät, Heinrich-Heine-Universität Düsseldorf, Düsseldorf 40225, Germany; Institut für Biologische Informationsprozesse, Strukturbiochemie (IBI-7), Forschungszentrum Jülich, Jülich 52425, Germany; Department of Biochemistry & Centre for Prions and Protein Folding Diseases, University of Alberta, Edmonton, AB T6G 2M8, Canada; Department of Biochemistry & Centre for Prions and Protein Folding Diseases, University of Alberta, Edmonton, AB T6G 2M8, Canada; Department of Biochemistry & Centre for Prions and Protein Folding Diseases, University of Alberta, Edmonton, AB T6G 2M8, Canada; Department of Biochemistry & Centre for Prions and Protein Folding Diseases, University of Alberta, Edmonton, AB T6G 2M8, Canada; Institut für Physikalische Biologie, Mathematisch-Naturwissenschaftliche Fakultät, Heinrich-Heine-Universität Düsseldorf, Düsseldorf 40225, Germany; Institut für Biologische Informationsprozesse, Strukturbiochemie (IBI-7), Forschungszentrum Jülich, Jülich 52425, Germany; Institut für Physikalische Biologie, Mathematisch-Naturwissenschaftliche Fakultät, Heinrich-Heine-Universität Düsseldorf, Düsseldorf 40225, Germany; Institut für Biologische Informationsprozesse, Strukturbiochemie (IBI-7), Forschungszentrum Jülich, Jülich 52425, Germany; Department of Biochemistry & Centre for Prions and Protein Folding Diseases, University of Alberta, Edmonton, AB T6G 2M8, Canada; Neuroscience and Mental Health Institute, University of Alberta, Edmonton, AB T6G 2M8, Canada; Institut für Physikalische Biologie, Mathematisch-Naturwissenschaftliche Fakultät, Heinrich-Heine-Universität Düsseldorf, Düsseldorf 40225, Germany; Institut für Biologische Informationsprozesse, Strukturbiochemie (IBI-7), Forschungszentrum Jülich, Jülich 52425, Germany

**Keywords:** α-synuclein, amyloid, conformation, disease, fibril, immunization, vaccine

## Abstract

The progressive age-related aggregation of soluble α-synuclein into toxic oligomers and insoluble amyloid fibrils causes Parkinson’s disease, Lewy body dementia and multiple system atrophy, all of which are neurodegenerative diseases without a cure. Because α-synuclein is a self-antigen, pathogenic α-synuclein aggregates do not elicit a strong immune response. Recent advances in structural biology elucidating the structure of α-synuclein fibrils have allowed us to design engineered protein fibrils that model conformational epitopes present on the surface of α-synuclein fibrils.

HET-s is a soluble fungal protein capable of forming amyloid fibrils. We used HET-s(218-298) fibrils and four modified derivatives, each displaying a selected conformational epitope present on the surface of α-synuclein fibrils, to vaccinate TgM83^+/−^ mice, a model for Parkinson’s disease-like synucleinopathies.

Fibrillar vaccine candidates significantly extended the survival of immunized TgM83^+/−^ mice by ≤38% after intraperitoneal challenge and ≤42% after intragastric challenge with α-synuclein fibrils. Fully immunized mice developed antibodies that recognized α-synuclein fibrils and brain homogenates from patients with dementia with Lewy bodies, multiple system atrophy and Parkinson’s disease.

Fibrillar vaccine candidates that mimic conformational epitopes on the surface of pathological α-synuclein fibrils have the ability to induce immunity and protection against Parkinson’s disease and other synucleinopathies.

## Teaser

Because α-synuclein is a self-antigen, pathogenic α-synuclein aggregates fail to elicit a robust immune response. Ma *et al.* show that vaccines based on modified fungal protein fibrils mimicking α-synuclein conformational epitopes extend survival in mouse models of Parkinson’s disease-like synucleinopathy.

## Introduction

Parkinson’s disease (PD), Lewy body dementia (LBD) and multiple system atrophy (MSA) are incurable synucleinopathies that are caused by the misfolding and aggregation of soluble α-synuclein (α-syn) protein into oligomers and insoluble amyloid fibrils that are toxic to neurons and cause progressive neurodegeneration with characteristic motor impairment and additional non-motor symptoms.^1-3^

Pathological accumulations of α-syn have prion-like properties in that they have the ability to propagate from afflicted to unaffected, healthy neurons, thereby seeding *de novo* aggregation of α-syn by recruiting its monomers into oligomers and growing fibrils. Subsequently, these growing fibrils undergo fragmentation, yielding smaller seed species that perpetuate this deleterious cycle. The transmission of pathological α-syn aggregates between neurons has been shown to occur via trans-synaptic routes, possibly mediated by various mechanisms, such as vesicular trafficking, receptor-mediated endocytosis, tunnelling nanotubes and simple diffusion.^4^ Investigations in both human subjects and animal models provide empirical support for the dissemination of α-syn pathology over large anatomical distances within the nervous system over time.^5,6^ The rostrocaudal spread of α-syn pathology within the CNS in PD is described by the Braak staging system.^6^ In addition, accumulating evidence underscores the ability of α-syn pathology to propagate from the enteric nervous system, which innervates the gastrointestinal tract, to the CNS, either via peripheral nerves or through the bloodstream.^7-11^

Until recently, synucleinopathies could be distinguished clinically based on their neurological presentation and, more precisely, neuropathologically, based on the cell types involved, which are neuronal in PD and LBD and mostly oligodendrocytic in MSA.^1,2,12,13^ Advances in the structural analysis of synthetic and pathological α-syn fibrils isolated from patient brains now show that differences in these diseases are encoded at the molecular level, with specific conformations of α-syn fibrils causing specific synucleinopathies.^14-17^ In PD and LBD, α-syn forms a single protofilament fibril with a Lewy-type fold, whereas in MSA, α-syn forms asymmetrically paired filaments with an MSA type I or II fold. These pathological α-syn fibrils, which are at the root of synucleinopathies, are a promising target for therapeutic intervention that could provide a cure or protection against these diseases.

**Figure 1:**
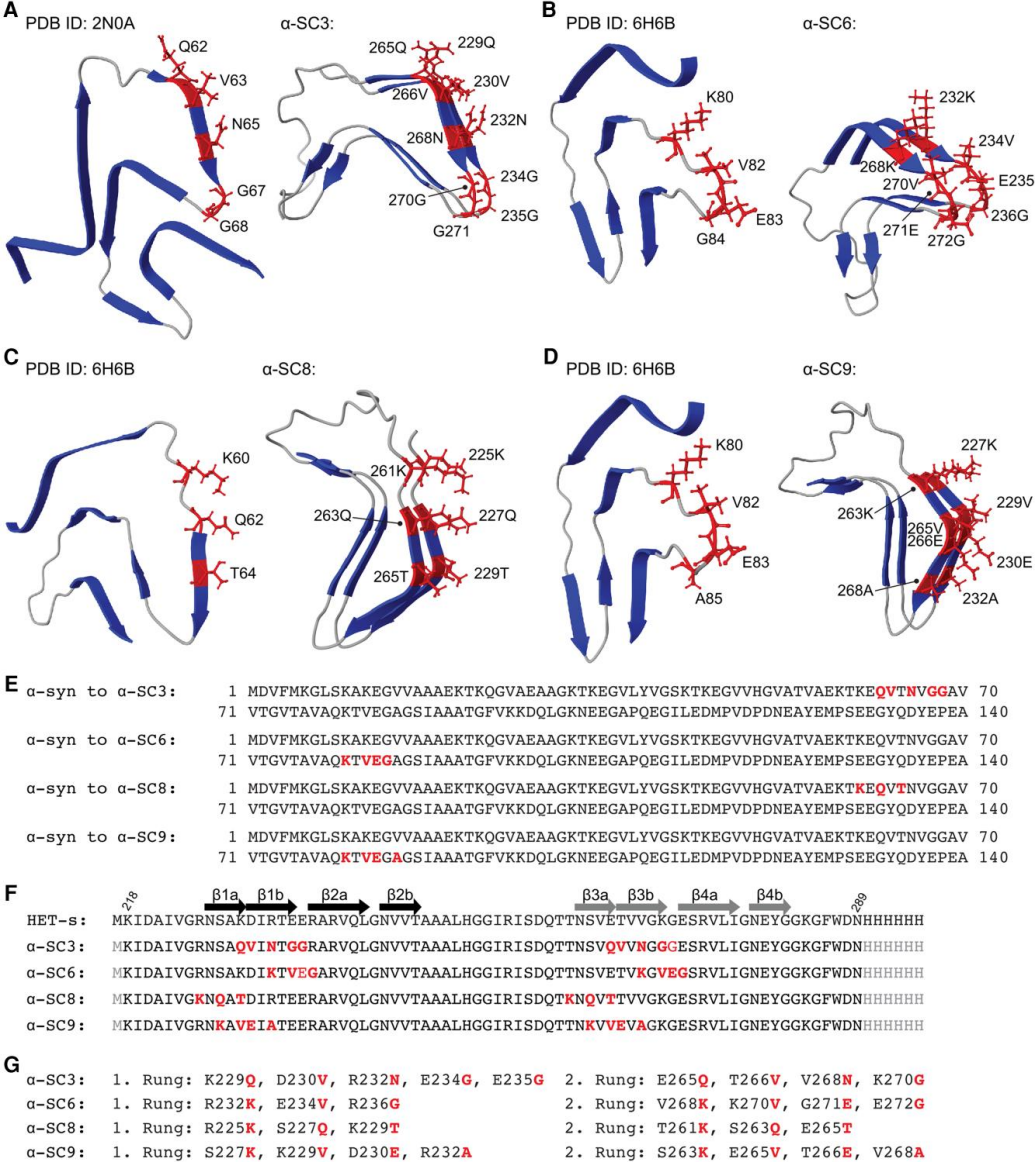
**HET-s-based vaccine candidates with conformational epitopes that mimic the surface of α-syn fibrils.** (**A**–**D**) Based on the structure of two synthetic α-syn fibrils, Protein Data Bank Identification codes (PDB IDs) 2N0A (**A**) and 6H6B (**B**–**D**), we identified selected amino acid residues (red) that form conformational epitopes on the surface of these fibrils.^14,15^ We mutated HET-s(218–289), PDB ID 2RNM, to express the selected amino acid residues (red) in a structurally similar context, yielding four different vaccine candidates: α-SC3, α-SC6, α-SC8 and α-SC9.^20^ (**E**) Primary structure of α-syn with selected amino acid residues (red) that form distinct conformational epitopes in α-syn fibrils introduced into HET-s(218–289) to yield vaccine candidates α-SC3, α-SC6, α-SC8 and α-SC9. (**F**) Primary structure of HET-s(218–289) and its derived vaccine candidates α-SC3, α-SC6, α-SC8 and α-SC9 showing amino acid residues (red) mimicking continuous surface epitopes in α-syn fibrils. In fibrillar HET-s(218–289), eight β strands form two coils in a left-handed β solenoid, with each coil having four β strands. (**G**) Mutations introduced into HET-s(218–289) to yield vaccine candidates α-SC3, α-SC6, α-SC8 and α-SC9.

Previously, we showed that an engineered quadrivalent vaccine mimicking α-syn fibrils induces an immune response against pathological α-syn fibrils by molecular grafting of conformational epitopes present on the surface of α-syn fibrils onto the inert carrier molecule HET-s(218–289) (PDB IDs: 2RNM), the prion domain of the HET-s protein found in the filamentous fungus *Podospora anserina*.^18^ Residues 218–289 in the C-terminus of HET-s are unstructured in solution and the non-prion state and can form biologically functional amyloid fibrils when the prion state is adopted.^19^ HET-s(218–289) fibrils are assemblies of four β-strands forming two coils of a left-handed β-solenoid.^20,21^ Based on the known structure of two synthetic α-syn fibrils (PDB IDs: 2N0A and 6H6B), we mutated selected amino acid residues in HET-s(218–289) to design four vaccine candidates α-SC3, α-SC6, α-SC8 and α-SC9 (Fig. 1), all of which form amyloid fibrils.^14,15,22^ Importantly, these vaccines were safe, because none of them seeded α-syn aggregation in seed amplification assays or in α-synA53T-YFP biosensor cells.^23^ We have shown in mouse models of PD that vaccination with a quadrivalent vaccine that combined α-SC3, α-SC6, α-SC8 and α-SC9 induced antibodies that recognize α-syn fibrils and protected against PD-like symptoms, resulting in improved motor performance and prolonged survival. Specifically, immunization with this quadrivalent vaccine resulted in a modest 8% increase in survival in a mouse model of brain-first PD after intracerebral challenge with α-syn fibrils, and a significant 21% and 22% increase in survival in two mouse models of body-first PD after intraperitoneal or intragastric challenge with α-syn fibrils, respectively.^23^

Here, we investigated the effect of each of the four vaccine candidates and HET-s fibrils individually on survival in two mouse models of body-first PD. We quadrupled the vaccine dose and demonstrated that each of the four vaccine candidates and HET-s fibrils, individually protected against weight loss and motor impairment and significantly prolonged survival by ≤42%.

## Materials and methods

### Animals

B6;C3-Tg(*Prnp*-SNCA*A53T)83Vle/J mice (TgM83^+/−^ mice) hemizygous for a transgene encoding human α-syn with the familial A53T mutation were acquired from The Jackson Laboratory and crossed with wild-type C57BL/6J mice to obtain hemizygous offspring. Animals were genotyped by real-time PCR. Group sizes were determined *a priori* to allow for the detection of significant differences in motor function and survival. No animals were excluded during the experiment. Animals were housed in the same location and randomly assigned to treatment groups based on birth and availability. The authors L.M., V.P., S.R. and G.T. were aware of the group assignments at all times. All studies involving animals were approved by the animal protection committee of the North Rhine-Westphalia State Environment Agency (LANUV). All applicable national and institutional guidelines for the care and use of animals were followed.

### Preparation of HET-s-based fibrils

DNA encoding HET-s, α-SC3, α-SC6, α-SC8 and α-SC9 had been subcloned into the pET-21a vector (Novagen) for expression in *Escherichia coli* BL21(DE3).^23^ Starter cultures were grown in 25 ml of 2YT medium at 37°C and 250 rpm overnight and were used to inoculate 500 ml of 2YT medium with 100 µg/ml ampicillin, which was grown to an optical density of 0.8 at 600 nm. The culture was cooled down to 25°C, induced with 1 mM isopropyl-β-D-1-thiogalactopyranoside (IPTG), and grown overnight at 37°C and 250 rpm. Cells were harvested at 6500*g* and 4°C for 25 min, and frozen for ≥30 min. Pellets were resuspended in 20 ml IB buffer (100 mM Tris-HCl, pH 8.0) with 0.5% Triton X-100, 1 mg/ml lysozyme (Sigma-Aldrich), and 1× complete EDTA-free protease inhibitor cocktail (Roche). After incubation at room temperature for 30 min, the lysed cells were sonicated (Sonifier 250; Branson Ultrasonics) for five cycles (1 min on and 1 min off) at an output voltage of 50 V with a 50% duty cycle at 4°C. Subsequently, 3 U/ml benzonase (Merck) was added for each 1 ml of the original culture, and the homogenates were incubated for 20 min, then centrifuged at 15 557*g* and 4°C for 30 min. The supernatants were discarded, and pellets containing the expressed proteins as inclusion bodies were frozen for ≥30 min. The pellets were resuspended in IB buffer containing 0.5% Triton X-100, 1 mg/ml lysozyme and 1× complete EDTA-free protease inhibitor cocktail, and were then incubated for 20 min at room temperature. After another sonication and centrifugation step as described above, the process was repeated twice and the homogenates centrifuged at 15 557*g*, 4°C for 30 min. Finally, the pellets containing purified inclusion bodies were resuspended in IB buffer and stored at −20°C. Inclusion bodies of recombinant proteins were solubilized in 6 M guanidine hydrochloride, 20 mM sodium phosphate, 0.5 M NaCl at pH 8.0, and stirred at room temperature for 45 min. The homogenates were clarified by ultracentrifugation at 235 000*g* for 35 min at 4°C. Recombinant proteins were purified in denaturing conditions by affinity chromatography using a BioLogic DuoFlow chromatography system (Bio-Rad Laboratories). HisTrap HP columns (GE Healthcare Life Sciences) were equilibrated with equilibrium buffer (8 M urea, 20 mM sodium phosphate, 0.5 M NaCl and 10 mM imidazole, pH 8.0) until the absorbance at 280 nm was stable. The columns were loaded with the clarified lysates, then washed with equilibrium buffer until the absorbance at 280 nm was stable again. Bound proteins were eluted using a linear gradient from 10 to 500 mM imidazole in equilibrium buffer. Peaks containing recombinant protein were automatically collected with a BioFrac fraction collector (Bio-Rad Laboratories) when the absorbance at 280 nm was >0.05. Subsequently, the buffer was exchanged to 175 mM acetic acid at pH 2.8 using HiTrap desalting columns (GE Healthcare Life Sciences). The purified, desalted and denatured proteins were fibrillized by increasing the pH to 7.5 with 3 M Tris base (pH 13.0) and stirring at 600 rpm at room temperature for 5 days. Prior to immunization, vaccine candidates were sonicated on ice using a Sonoplus Mini20 (Bandelin) ultrasonic homogenizer equipped with an MS 1.5 microtip. Samples were subjected to a single 12 s pulse at 50% amplitude to increase the uniformity of the inoculum.

### Vaccinations and plasma collection

Adult mice (*n* = 10–12 per group) at an age of 6–8 weeks were anaesthetized with 2%–3% isoflurane and injected intraperitoneally with 200 µl of 100 µg antigen, composed of either HET-s, α-SC3, α-SC6, α-SC8 or α-SC9 fibrils, diluted 1:1 in alum (Alhydrogel adjuvant 2%, InvivoGen) using a 26-gauge needle. Each animal was given four vaccine injections, each spaced 2 weeks apart. Blood samples of 50 µl were obtained from the tail vein of each mouse immediately before each injection and 2 weeks after the final injection. These samples were diluted 1:1 with 5% sodium citrate. The blood was then centrifuged at 500*g* for 10 min at 4°C, and the resulting plasma was collected and stored at −80°C.

### Synthesis of α-syn monomers and fibrils

N-Terminally acetylated human wild-type α-synuclein was expressed in *E. coli* BL21(DE3) cells containing the pT7 vector for codon-optimized α-synuclein and the pNatB vector for the N-terminal acetyltransferase B complex from *Schizosaccharomyces pombe*. The bacteria were grown overnight in 120 ml of lysogeny broth (LB) medium with 100 µg/ml ampicillin and 34 µg/ml chloramphenicol at 37°C and 120 rpm. The next day, the optical density at 600 nm was measured, and the culture was diluted to an optical density of 0.1 in 1 l of LB medium. The culture was incubated with 100 µg/ml ampicillin and 34 µg/ml chloramphenicol at 37°C until the optical density reached 1.0–1.2. Expression was induced with 1 mM IPTG. After 4.5 h, the cells were pelleted at 5000*g* and 4°C. The pellets were resuspended in 20 mM Tris (pH 8.0) containing a protease inhibitor (Roche) and boiled for 2 × 15 min, followed by centrifugation at 20 000*g* and 4°C for 30 min. Ammonium sulfate precipitation was performed by adding 0.45 g/ml of (NH_4_)_2_SO_4_ crystals to the supernatant over 5 min and stirring for 15 min. After centrifugation, the pellet was resuspended in 50 ml of 20 mM Tris-HCl (pH 8.0). α-Syn was purified using a HiPrep QFF 16/10 anion exchange chromatography column with a linear gradient from 20 mM Tris-HCl (pH 8.0) to 1 M NaCl in 20 mM Tris-HCl (pH 8.0) on an ÄKTA pure chromatography system (GE Healthcare). The ammonium sulfate precipitation was repeated, and the pellet was resuspended in 50 mM Tris-HCl (pH 7.2). Further purification was carried out using a HiLoad 16/60 Superdex 75 pg size exclusion column (Cytiva) over 1.5 column volumes. NaCl was added to achieve a final concentration of 50 mM Tris-HCl and 150 mM NaCl. The protein was concentrated to 5 mg/ml using a Vivaspin concentrator (Sartorius). The α-synuclein monomer was fibrillized by incubation at 37°C and 1000 rpm on a Thermomixer (Eppendorf) for 7 days. Fibrils were sonicated in four 15-s steps with 2 min pauses between each step using a Sonoplus Mini20 (Bandelin) and an MS 1.5 microtip.

### Challenge of TgM83^+/−^ mice with α-syn fibrils

For intraperitoneal challenge, 50 μg of sonicated α-syn fibrils in 20 μl PBS were injected into the peritoneum of TgM83^+/−^ mice (*n* = 11–12 per group) anaesthetized with isoflurane, using a 30-gauge disposable hypodermic needle. For gut wall injections of α-syn fibrils in TgM83^+/−^ mice (*n* = 10–12 per group), an aseptic laparotomy was performed. The mice received a subcutaneous injection of buprenorphine (0.05 mg/kg, Bayer) for analgesia, followed by anaesthesia with isoflurane. The abdomen was shaved, disinfected, and a small incision was made with a scalpel. α-Syn fibrils were injected into the wall of the pylorus and duodenum at four points, spaced 0.5 cm apart, using a 10 µl Hamilton syringe. Each site received an injection of 6.25 µg (2.5 µl) of α-syn fibrils. The abdominal wall was sutured, and the skin was closed with wound clips, which were removed 2 weeks later. After surgery, the animals received a single subcutaneous dose of carprofen (5 mg/kg) and were provided with a mix of metamizole (0.5 mg/ml, WDT) and 10% (w/v) sucrose in their drinking water for 3 days. The animals were monitored daily for general health and three times a week for signs of neurological disease, including reduced grooming, ataxia, tremor, bradykinesia, akinesia, lethargy, circling, tail rigidity, paraparesis, paralysis, kyphosis and other symptoms. Body weight was recorded weekly. For immunohistochemical analysis, diseased mice were euthanized with ketamine/xylazine and underwent transcardial perfusion with PBS followed by 4% formalin (Sigma) in PBS. The brains were dissected and stored in 4% formalin in PBS for later processing. For biochemical analysis, diseased mice were euthanized by cervical dislocation, and the brains were snap-frozen on dry ice and stored at −80°C for later processing. Statistical analysis was performed using GraphPad Prism version 10.

### Extraction of α-synuclein filaments

Sarkosyl-insoluble material was extracted from fresh-frozen caudate putamen of individuals with PD, DLB and MSA. Briefly, tissues were homogenized on ice in 20 volumes (v/w) of extraction buffer containing 10 mM Tris-HCl (pH 7.5), 0.8 M NaCl, 10% sucrose and 1 mM EGTA. Homogenates were adjusted to 2% sarkosyl and incubated for 1 h at 37°C. Following centrifugation at 10 000*g* for 10 min at 4°C, the supernatants were centrifuged at 100 000*g* for 60 min at 4°C. Resulting pellets were resuspended in 1 ml/g extraction buffer and centrifuged at 5000*g* for 5 min at 4°C. Supernatants were then diluted 3-fold in 50 mM Tris-HCl (pH 7.5) containing 0.15 M NaCl, 10% sucrose and 0.2% sarkosyl, and centrifuged at 100 000*g* for 30 min at 4°C. Final sarkosyl-insoluble pellets were resuspended in 100 μl/g of 20 mM Tris-HCl (pH 7.4) containing 50 mM NaCl.

### Immunoprecipitations

Plasma samples of 50 µl were incubated with 16.7 µl magnetic Dynabeads Protein G (Invitrogen) for 1 h with rotation at room temperature. The conformation-specific anti-α-syn aggregate antibody MJFR-14-6-4-2 (Abcam) served as a positive control, and 0.625 µg was incubated with 16.7 µl of magnetic beads in 50 µl of PBS and 0.02% Tween 20 for 1 h with rotation at room temperature. The beads were washed three times with 50 µl of PBS, resuspended in 49.5 µl of 1× lysis buffer, supplemented with 0.5 µl of extracted α-synuclein filaments, and incubated overnight with rotation at 4°C. After magnetic pull-down of the beads, cleared supernatants of the extracts were used for further analysis.

### Time-resolved fluorescence resonance energy transfer

Quantification of α-syn filaments in sarkosyl extracts of brain homogenates before and after immunoprecipitations was performed using the HTRF α-synuclein aggregation detection kit (Revvity) for time-resolved fluorescence resonance energy transfer (TR-FRET) measurements. Briefly, 10 μl samples of the cleared extracts were supplemented with 10 µl of a premixed antibody solution containing anti-h-α-Synuclein-d2 (acceptor) and anti-h-α-Synuclein-Tb-Cryptate (donor). Samples were transferred to an HTRF 96-well low-volume plate (Revvity), covered with a plate sealer, and incubated for 20 h at room temperature. Fluorescence emission was measured at 665 nm for FRET-dependent acceptor fluorescence and at 620 nm for FRET-independent donor fluorescence using a CLARIOstar microplate reader (BMG Labtech). The ratio of both fluorescence emission values, multiplied by 10 000, is directly proportional to the amount of human α-synuclein oligomers and aggregates in each sample. The Δ*F* (%) value, a measure of the signal-to-background ratio, was calculated by dividing the difference between the ratio of the sample and the ratio of the negative control by the ratio of the negative control, then multiplying the result by 100. GraphPad Prism version 10 was used for statistical analysis.

### ELISA

In brief, 96-well high-binding ELISA plates (Corning) were coated overnight at 4°C with 100 µl per well of a 5 µg/ml solution of sonicated fibrils, with gentle agitation. The plates were then washed twice with PBS containing 0.1% Tween 20 (PBST) and once with PBS. They were blocked with 200 µl per well of a blocking buffer [5% (w/v) milk powder in PBS] and washed again five times with PBST and once with PBS. Next, 100 µl of serum was added to each well, and the plates were incubated overnight at 4°C. To detect the vaccine candidates or HET-s fibrils, plasma from one mouse collected at five different time points was tested at a 1:330 000 dilution in PBS. To detect α-syn fibrils, both preimmune and immune plasma samples were diluted 1:1000 in PBS. After five washes with PBST and one with PBS, the plates were incubated for 2 h at room temperature with 100 µl per well of an anti-mouse horseradish peroxidase-linked secondary antibody (Invitrogen) at a 1:5000 dilution. Following five washes with PBST and one with PBS, 100 µl per well of TMB substrate solution (Pierce TMB Substrate Kit, Thermo Fisher Scientific) was added. The colour reaction was stopped after 50 min with 2 M sulfuric acid, and the absorbance was measured at 450 nm using a CLARIOstar microplate reader (BMG Labtech).

### Competitive ELISA

A competitive ELISA protocol was used to assess the recognition of α-syn aggregates in patient brain homogenates by the plasma of fully immunized mice. A 96-well high-binding ELISA plate (Corning) was coated overnight at 4°C with 100 µl per well of a 5 µg/ml solution of sonicated HET-s-derived fibrils, with gentle agitation. The plate was then blocked with 3% bovine serum albumin in PBS for 90 min and washed twice with PBST and once with PBS. Separately, on a 96-well low-binding cell culture plate, 75 µl per well of a 1:330 000 dilution of serum was mixed with 66.67 µg/ml of brain homogenate [DLB, MSA, PD, or non-neurological (healthy) control] and incubated overnight at 4°C. Next, 100 µl per well of the mixture was transferred to the fibril-coated and blocked ELISA plates and incubated overnight at 4°C. Afterwards, the plates were washed twice with PBST and once with PBS, followed by the addition of 100 µl per well of an anti-mouse horseradish peroxidase-linked secondary antibody at a 1:5000 dilution and incubation at room temperature for 2 h. The plates were then washed four times with PBST and once with PBS. Subsequently, 100 µl per well of TMB substrate solution was added. The colour reaction was stopped after 30 min with 2 M sulfuric acid, and absorbance was measured at 450 nm using a CLARIOstar microplate reader.

### Grip strength analysis

Unvaccinated and vaccinated animals were tested at 3 and 7 months after challenge.^23^ To measure grip strength, mice were allowed to grasp a grid attached to a grip strength meter (Ugo Basile) with all four paws. They were then gently pulled by the tail until they released the grid. Grip strength was measured three times, with 15 min breaks between each test. The average of the three trials was recorded as the performance of the animal for that day. Relative grip strength was calculated as the percentage of the mean performance at the later time point compared with the performance at the earlier time point. GraphPad Prism version 10 was used for statistical analysis. Outliers were identified and removed using the ROUT function with Q set to 2%.

## Results

### Vaccination prolongs survival in two body-first mouse models of Parkinson’s disease

**Figure 2:**
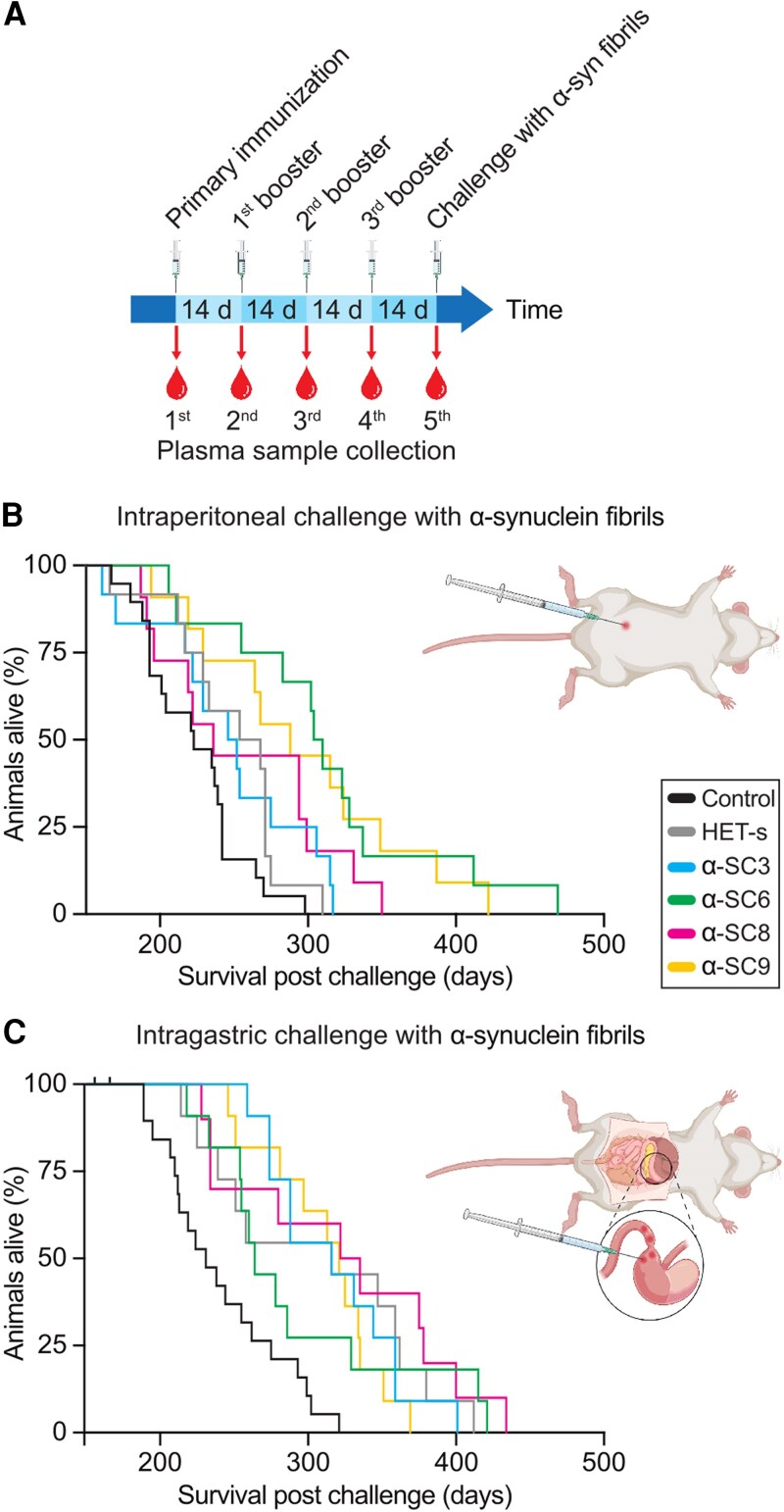
**Vaccination prolongs survival of TgM83^+/−^ mice after challenge with α-syn fibrils.** (**A**) Groups of TgM83^+/−^ mice were immunized intraperitoneally with either HET-s fibrils or one of its four engineered derivatives, α-SC3, α-SC6, α-SC8 or α-SC9 fibrils, every 2 weeks, with a total of four doses of 100 µg each. Blood plasma was collected once before and every 2 weeks after administration of each vaccine candidate. To model body-first PD, mice were injected with α-syn fibrils into the peritoneum (**B**) or the intestinal wall (**C**). (**B**) In intraperitoneally challenged mice, vaccination prolonged median survival from 223 days in non-immunized control mice to 261 days (*P* < 0.05) for HET-s fibrils, 249 days (*P* < 0.05) for α-SC3 fibrils, 307 days (*P* < 0.001) for α-SC6 fibrils, 236 days (n.s.) for α-SC8 fibrils and 288 days (*P* < 0.01) for α-SC9 fibrils in fully immunized mice. (**C**) In mice injected with α-syn fibrils into the intestinal wall, vaccination prolonged median survival from 231 days in unimmunized control mice to 316 days (*P* < 0.01) for HET-s fibrils, 316 days (*P* < 0.001) for α-SC3 fibrils, 264 days (*P* < 0.05) for α-SC6 fibrils, 328.5 days (*P* < 0.01) for α-SC8 fibrils and 321 days (*P* < 0.001) for α-SC9 fibrils in fully immunized mice. Survival was analysed using Kaplan–Meier curves and the log-rank (Mantel–Cox) test. Figure partially created in BioRender. Tamgüney, G. (2025) https://BioRender.com/qyi8gmt.

Hemizygous TgM83^+/−^ mice expressing human α-syn with the familial A53T mutation develop PD-like neuropathology and disease when injected with synthetic or patient-derived α-syn fibrils. In contrast, unchallenged TgM83^+/−^ mice remain free of neurological disease and neuropathology at least up to an age of 650 days.^10,23-26^ To determine whether HET-s-derived vaccine candidates mimicking α-synuclein fibrils elicit immunity to PD and related synucleinopathies and delay the onset of neurological disease, we vaccinated separate groups of adult TgM83^+/−^ mice via intraperitoneal injections. In a previous study, we had vaccinated animals with a quadrivalent mixture of α-SC3, α-SC6, α-SC8 and α-SC9 fibrils (Fig. 1).^23^ In contrast to our previous study, here we vaccinated groups of animals with either unmodified HET-s fibrils or with only one of the four HET-s-derived fibrillar vaccine candidates, α-SC3, α-SC6, α-SC8 or α-SC9, to assess the level of protection conferred by each vaccine candidate individually (Fig. 1 and Supplementary Fig. 1). Furthermore, in contrast to our previous study, in which we used 25 µg doses of each vaccine, we used 100 µg doses here, representing a 4-fold increase in the dose administered.^23^ The animals were vaccinated at 2-week intervals for a total of four times (Fig. 2A). Unvaccinated mice injected with α-syn fibrils from our previous study also served in this study as controls, because all experiments were initiated at approximately the same time.^23^ To analyse the vaccine-induced antibody response of TgM83^+/−^ mice, we collected plasma once before and every 2 weeks after each vaccine dose was administered (Fig. 2A).

Recent findings suggest that PD might originate in either the CNS (brain-first PD) or the enteric nervous system (body-first PD), followed by a prion-like spread of pathology either from the brain to the body or from the body to the brain, respectively, resulting in an end-stage pathology that is not well distinguishable between the two subtypes of PD.^27^ In this study, we modelled body-first PD by challenging fully vaccinated TgM83^+/−^ mice with injections of synthetic α-syn fibrils either into the peritoneum or into the wall of the stomach and pylorus, which seeds α-syn aggregation in the enteric nervous system first. Biologically, the two routes model distinct stages of peripheral disease: the intragastric route recapitulates the initial emergence of seeds within the enteric nervous system, whereas the intraperitoneal route reflects the dissemination of seeds from the enteric nervous system into the broader peripheral compartment. For intraperitoneally challenged mice, immunization with all vaccine candidates, with the exception of α-SC8 fibrils (236 days, *P* = 0.063), significantly prolonged survival in comparison to unvaccinated control mice with a median survival of 223 days (Fig. 2B and Table 1). Survival was most prolonged, by 38%, in mice immunized with α-SC6 fibrils (307 days, *P* < 0.0001). It is noteworthy that vaccination with unmodified HET-s fibrils (261 days, *P* < 0.05) also significantly protected mice from disease upon challenge, with a slightly more pronounced effect than that observed with α-SC3 fibrils (249 days, *P* < 0.05). We observed the most effective protection of vaccination in mice challenged with α-syn fibrils via the intragastric route (Fig. 2C and Table 1). In comparison to unvaccinated mice with a median survival of 231 days, vaccination with α-SC8 fibrils (328.5 days, *P* < 0.01) and α-SC9 fibrils (321 days, *P* < 0.001) prolonged survival by 42% and 39%, respectively. Furthermore, vaccination with α-SC3 fibrils (316 days, *P* < 0.001) and HET-s fibrils (316 days, *P* < 0.01) resulted in a 37% increase in survival.

**Table 1:** Survival data

Route of challenge	Unvaccinated control^23^	Quadrivalent vaccine^23^	HET-s	α-SC3	α-SC6	α-SC8	α-SC9
Intraperitoneal							
Censored animals (*n*)	0	0	0	0	0	0	0
Animals (*n*)	19	11	12	12	12	11	11
Median survival (days)	223	271	261	249	307	236	288
Survival extension (%)	N.A.	22	17	12	38	6	29
* P* (log-rank)	N.A.	<0.01	<0.05	<0.05	<0.0001	n.s.	<0.01
Intragastric							
Censored animals (*n*)	1	0	1	0	1	0	0
Animals (*n*)	19	10	11	11	11	10	11
Median survival (days)	231	279.5	316	316	264	328.5	321
Survival extension (%)	N.A.	21	37	37	14	42	39
* P* (log-rank)	N.A.	<0.05	<0.01	<0.001	<0.05	<0.01	<0.001

N.A. = not applicable; n.s. = not significant.

### Vaccination protects against weight loss and decline in motor performance

**Figure 3:**
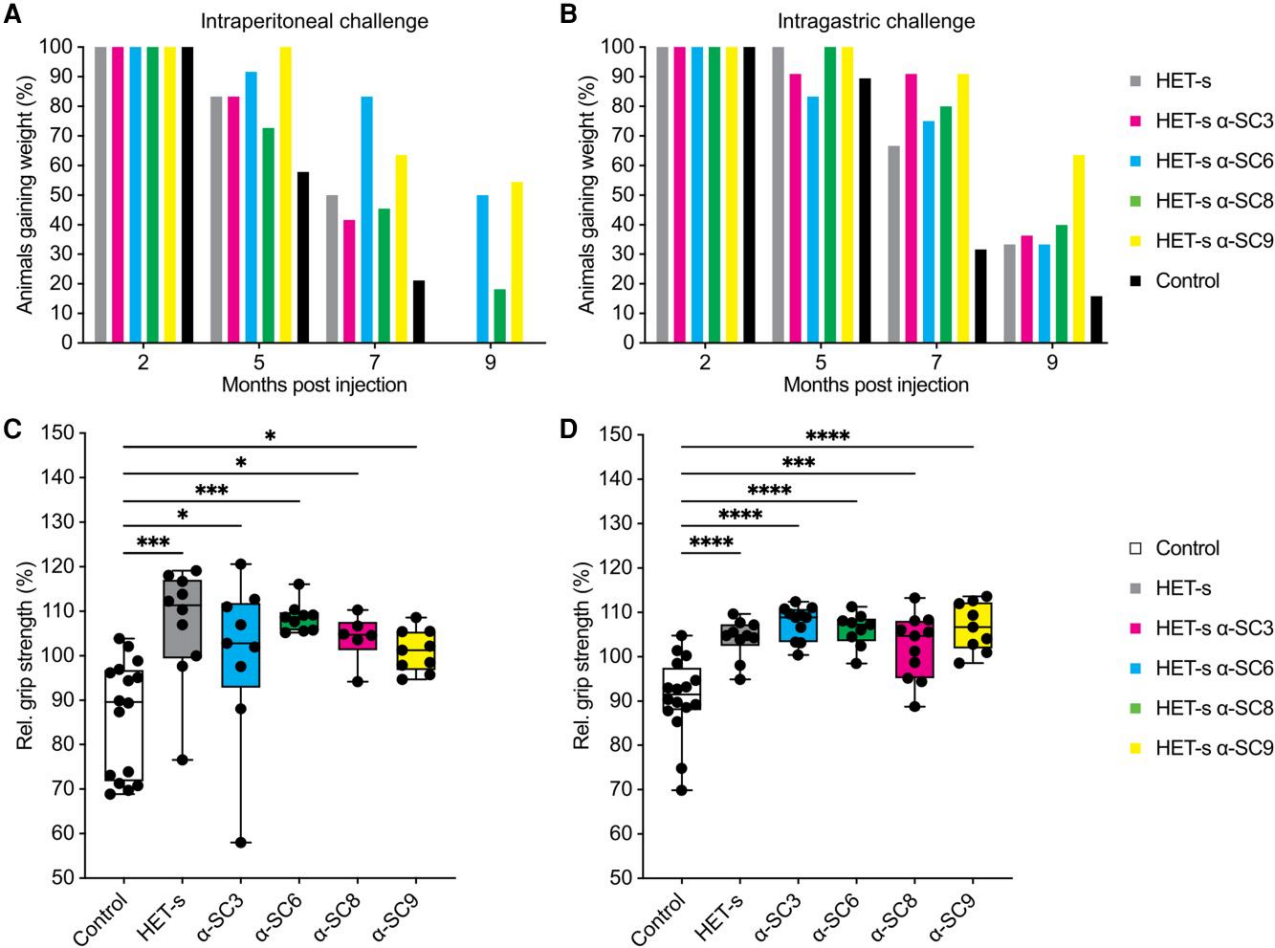
**Vaccination protects TgM83^+/−^ mice challenged with α-syn fibrils against early weight loss and motor deficits.** (**A** and **B**) All TgM83^+/−^ mice challenged with α-syn fibrils initially gained weight before they began to lose weight and develop neurological disease. The percentage of animals that gained weight at 2, 5, 7 and 9 months following intraperitoneal challenge (**A**) and following intragastric challenge (**B**) is shown. In both models of body-first PD, animals immunized with HET-s (grey), α-SC3 (magenta), α-SC6 (blue), α-SC8 (green) or α-SC9 (yellow) fibrils gained weight for longer periods of time than non-immunized controls (black). (**C** and **D**) Grip strength of immunized and non-immunized TgM83^+/−^ mice was assessed at 3 and 7 months following challenge with α-syn fibrils, as an indicator of motor performance. In animals injected intraperitoneally (**C**) or into the gut wall (**D**) with α-syn fibrils, the relative grip strength of animals immunized with HET-s (grey), α-SC3 (magenta), α-SC6 (blue), α-SC8 (green) or α-SC9 (yellow) fibrils was significantly higher than that of non-immunized control animals (white). Significance was assessed by one-way ANOVA followed by Dunnett’s multiple comparison test. Boxes represent 25th to 75th percentiles. Lines in the centre of the boxes indicate the median. Whiskers indicate minimum and maximum values. *P < 0.05, ***P < 0.001 and ****P < 0.0001.

Weight loss and a decline in motor performance are early surrogate markers of neurological disease, which we monitored in TgM83^+/−^ mice challenged with α-syn fibrils. After challenge with α-syn fibrils, TgM83^+/−^ mice exhibited a period of continued weight gain before entering a phase of weight loss (Supplementary Fig. 2), followed by the development of neurological disease. In both body-first models of PD, immunization with any of the four HET-s-derived vaccine candidates and with HET-s fibrils protected immunized mice from early weight loss (Fig. 3A and B). All TgM83^+/−^ mice that were injected with α-syn fibrils into the peritoneum or intestinal wall exhibited continued weight gain at 2 months post-challenge. We selected 5, 7 and 9 months post-challenge to visualize that immunization with all vaccine candidates and HET-s fibrils protected against early weight loss. In mice challenged intraperitoneally, α-SC6 and α-SC9 fibrils demonstrated the most efficacious protection (Fig. 3A). In mice injected with α-syn fibrils into the intestinal wall, all vaccine candidates, including HET-s fibrils, conferred comparable protection against weight loss, with α-SC9 fibrils being more protective at 9 months post-challenge (Fig. 3B).

As an indicator of motor performance, we measured the grip strength of immunized and non-immunized TgM83^+/−^ mice at 3 and 7 months after challenge with α-syn fibrils and calculated the relative grip strength (Fig. 3C and D and Table 2). In animals injected intraperitoneally with α-syn fibrils, all immunized animals exhibited a significantly higher relative grip strength (100%–109%) than non-immunized animals (86%). Likewise, in animals injected with α-syn fibrils into the intestinal wall, the relative grip strength of immunized animals (102%–108%) was also significantly greater than that of non-immunized animals (91%). In conclusion, following challenge with α-syn fibrils, immunized animals demonstrated a sustained increase in grip strength throughout the disease process, indicating a vaccine-induced protection and improvement in motor function.

**Table 2:** Grip strength data

Route of challenge	Vaccine candidate	Mean relative grip strength (%)	*P*-value
Intraperitoneal	N.A.	86	N.A.
	HET-s fibrils	107	<0.001
	α-SC3 fibrils	100	<0.05
	α-SC6 fibrils	109	<0.001
	α-SC8 fibrils	104	<0.05
	α-SC9 fibrils	101	<0.05
Intragastric	N.A.	91	N.A.
	HET-s fibrils	104	<0.0001
	α-SC3 fibrils	108	<0.0001
	α-SC6 fibrils	106	<0.0001
	α-SC8 fibrils	102	<0.001
	α-SC9 fibrils	107	<0.0001

N.A. = not applicable.

### Vaccination reduces the rate of degeneration in the CNS

We confirmed the presence of neurological disease in the brainstem of diseased mice by immunohistochemistry with an antibody to α-syn phosphorylated at serine 129 (Supplementary Fig. 3), which demonstrated the presence of deposits of pathological α-syn in neurons. We observed by immunofluorescence staining for α-syn phosphorylated at serine 129 and for ionized calcium-binding adaptor molecule 1 (Iba1) that neurons harbouring large deposits of pathological α-syn in the brainstem were surrounded by activated microglia (Supplementary Fig. 4A). Likewise, we detected by immunofluorescence staining for α-syn phosphorylated at serine 129 and for glial fibrillary acidic protein (GFAP) the recruitment of numerous astrocytes to the vicinity of neurons with α-syn deposits (Supplementary Fig. 4B). The findings demonstrate that the aggregation of α-syn in neurons of diseased mice had resulted in a neuroinflammatory response, as indicated by microgliosis and astrogliosis in brain areas with neuronal α-syn aggregation and deposition. Furthermore, we confirmed the presence of pathological α-syn aggregates in brain homogenates of diseased mice also by biochemical analysis (Supplementary Fig. 5). To quantify the differences in the amount of accrued neuropathology in the brains of vaccinated and unvaccinated mice, we measured α-syn aggregates in brain homogenates of sick mice by TR-FRET (Supplementary Fig. 6). The amount of brain-associated α-syn aggregates in sick mice was not significantly different regardless of their immunization status, whether vaccinated or not, or the route of challenge, intraperitoneal (Supplementary Fig. 6A) or intragastric (Supplementary Fig. 6B). In conclusion, administration of α-syn fibrils via injections into the peritoneum or the wall of the gastrointestinal tract seeded a progressive synucleinopathy in all challenged mice, regardless of their immune status. Within each group, all mice exhibited signs of neurological disease when comparable amounts of α-syn aggregates were present in the brain. This suggests that vaccination protected mice and enabled their longer survival by slowing down the accumulation of detrimental amounts of α-syn aggregates in the brain.

### Vaccination induces a robust immune response to the vaccine candidates

**Figure 4:**
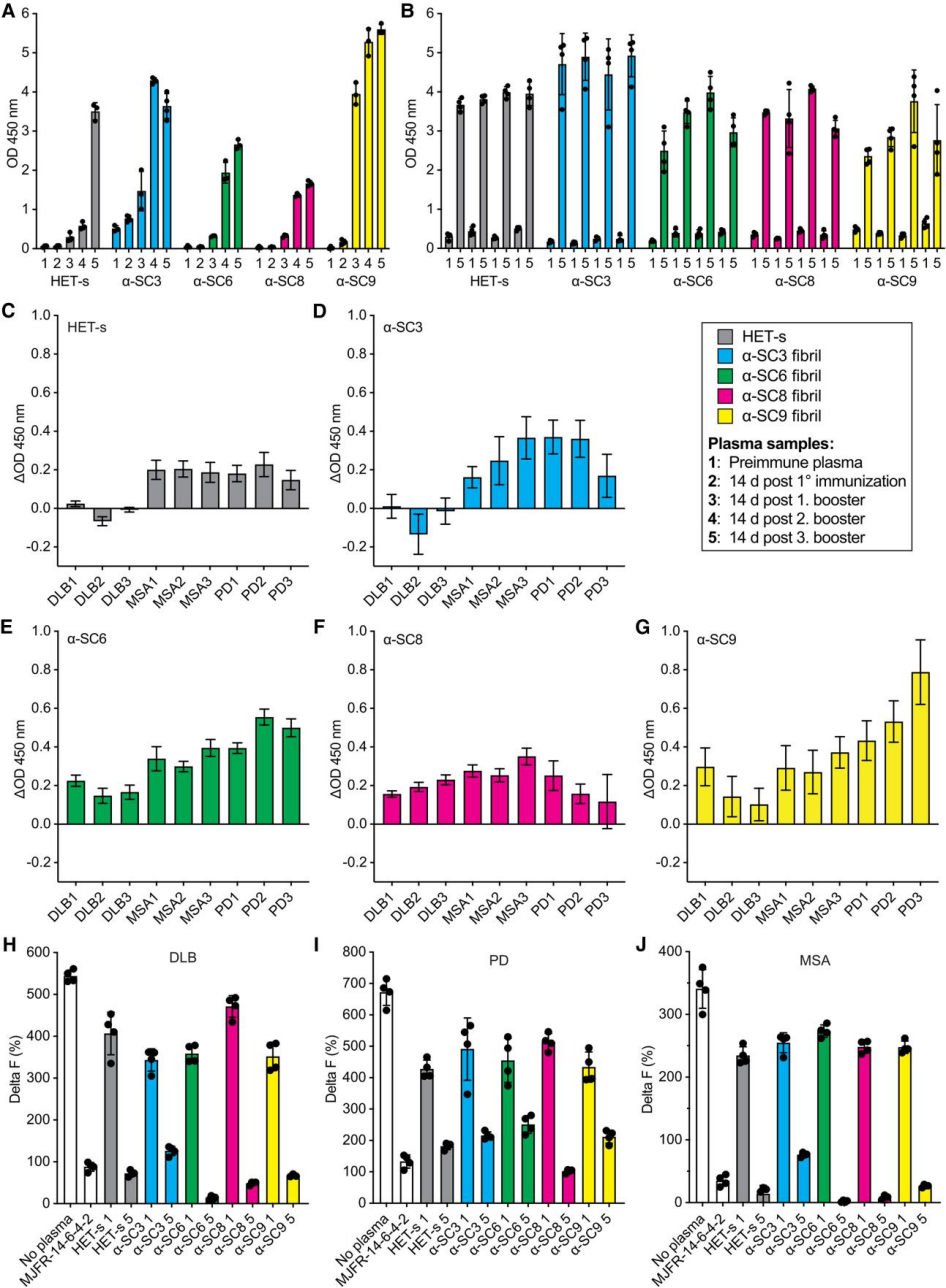
**Vaccination of TgM83^+/−^ mice stimulates the production of antibodies against pathological α-syn.** (**A**) ELISA measurements were conducted using blood plasma from three or four mice collected before (preimmune plasma) and 2 weeks after the primary immunization and each booster on ELISA plates coated with the original antigen. The results demonstrate that immunization with HET-s fibrils or any of the four HET-s-derived vaccine candidates progressively induced antibodies against the fibrillar antigens. (**B**) ELISA measurements were conducted using preimmune plasma and plasma from fully vaccinated mice (*n* = 4 individual mice per group). The results indicate that vaccination with HET-s fibrils or any of the four HET-s-derived vaccine candidates induced antibodies that recognize synthetic α-syn fibrils. (**C**–**G**) Competitive ELISA measurements were conducted using blood plasma from mice (*n* = 4 per group) fully immunized with HET-s fibrils (**C**), α-SC3 fibrils (**D**), α-SC6 fibrils (**E**), α-SC8 fibrils (**F**) or α-SC9 fibrils (**G**), and brain homogenates from five non-neurological (healthy) controls (used as a negative control) and three patients each with DLB, MSA or PD. The results indicate that vaccination elicited a highly variable degree of antibody response to pathological α-syn aggregates in the brains of patients. The response level varied depending on the vaccine candidate and the corresponding synucleinopathy, and was not observed in the case of blood plasma from mice immunized with HET-s or α-SC3 fibrils when tested against brain homogenates from DLB patients. The error bars indicate the mean ± standard error of the mean. (**H**–**J**) TR-FRET measurements were conducted on α-syn aggregates in sarkosyl-insoluble fractions obtained from patient brains with DLB (**H**), PD (**I**) and MSA (**J**) before and after immunoprecipitation using magnetic beads coupled to antibodies in blood plasma from fully immunized mice (*n* = 4 mice per group). The results indicate that HET-s fibrils and all four of its derivatives had induced antibodies that recognized pathological α-syn aggregates in brains of patients with synucleinopathies. Error bars indicate the mean ± standard deviation. DLB = dementia with Lewy bodies; ELISA = enzyme-linked immunosorbent assay; MSA = multiple system atrophy; OD = optical density; PD = Parkinson’s disease; TR-FRET = time-resolved fluorescence resonance energy transfer.

We analysed plasma samples collected from non-immunized and immunized mice via an ELISA and demonstrated that the primary vaccination and three subsequent booster vaccinations had induced a progressive increase in the antibody titres recognizing the HET-s, α-SC3, α-SC6, α-SC8 and α-SC9 fibrils that were used for immunization (Fig. 4A). The four vaccine candidates exhibited varying degrees of immunogenicity, as did the unmodified HET-s fibrils, resulting in a 3-fold difference in the magnitude of the induced antibody titres (α-SC9 > α-SC3 = HET-s > α-SC6 > α-SC8). The α-SC9 fibrils induced relatively high antibody titres following the first booster, whereas HET-s fibrils induced a strong response only following the final booster.

### Vaccination induces the production of antibodies that recognize pathological α-synuclein

To ascertain whether immunization had induced antibodies to synthetic α-syn fibrils, we used ELISA to measure the presence of antibodies in plasma samples from mice that had been immunized and those that had not (Fig. 4B). We observed that each of the four HET-s-derived vaccine candidates, in addition to HET-s fibrils, induced antibodies that recognized synthetic α-syn fibrils. The immune responses induced by HET-s, α-SC6, α-SC8 and α-SC9 fibrils were comparable to each other. Nevertheless, antibodies induced by α-SC3 fibrils demonstrated a slight superiority in recognizing synthetic α-syn fibrils when compared with those induced by α-SC6 (*P* < 0.01), α-SC8 (*P* < 0.01) and α-SC9 fibrils (*P* < 0.001).

### Vaccination induces antibodies that recognize pathological α-synuclein in patient brain homogenates

In a competitive ELISA (Supplementary Fig. 7), we tested antibodies in plasma from mice immunized with HET-s (Fig. 4C), α-SC3 (Fig. 4D), α-SC6 (Fig. 4E), α-SC8 (Fig. 4F) and α-SC9 fibrils (Fig. 4G) for their ability to recognize pathological α-syn in brain homogenates from patients with DLB (*n* = 3), MSA (*n* = 3) or PD (*n* = 3) when tested against five brain homogenates from non-neurological (healthy) controls (Supplementary Table 1). It is noteworthy that HET-s and α-SC3 fibrils did not appear to induce antibodies against α-syn aggregates present in the brains of patients with DLB. The α-SC3 fibrils induced a mild (ΔOD_450 nm_ = 2) to intermediate (ΔOD_450 nm_ = 4) antibody response, and the HET-s fibrils induced only a mild response to α-syn aggregates in the brains of patients with MSA and PD. The α-SC6 and α-SC9 fibrils induced a robust (ΔOD_450 nm_ ≥ 4) immune response to α-syn aggregates in the brains of patients with PD, a mild to intermediate response to α-syn aggregates in the brains of patients with MSA, and a mild response to those in the brains of patients with DLB. The α-SC8 fibrils exhibited a mild to intermediate response to α-syn aggregates in the brains of patients with MSA, and a mild response to α-syn aggregates in the brains of patients with DLB and PD.

To characterize the immune response induced by HET-s fibrils and each HET-s-derived vaccine candidate further, we prepared sarkosyl-insoluble fractions enriched in α-syn fibrils from brain homogenates of patients with DLB, PD and MSA (Supplementary Table 2).^16,17^ Subsequently, we conjugated the antibodies in plasma from non-immunized and fully immunized mice to protein G-coated magnetic beads to immunoprecipitate the *ex vivo* α-syn fibrils and measured the amount of α-syn fibrils remaining in the cleared fibril fractions using TR-FRET analysis (Fig. 4H–J). The magnetic beads without bound antibodies served as the negative control, whereas the magnetic beads coupled to the MJFR-14-6-4-2 antibody to α-syn served as the positive control. We observed that antibodies induced by all four vaccine candidates, α-SC3, α-SC6, α-SC8 and α-SC9 fibrils, in addition to HET-s fibrils, were capable of recognizing and immunoprecipitating *ex vivo* α-syn fibrils. The immunoprecipitation of α-syn fibrils purified from the brains of patients with DLB (Fig. 4H) and MSA (Fig. 4J) was slightly less efficient with plasma from mice immunized with α-SC3 fibrils than with plasma from mice immunized with α-SC6, α-SC8, α-SC9 and HET-s fibrils. The order of efficiency was α-SC6 > α-SC8 > α-SC9 = HET-s > α-SC3. The plasma from mice immunized with α-SC8 fibrils was slightly more efficient in immunoprecipitating α-syn fibrils purified from the brain of a patient with PD compared with the other three vaccine candidates and HET-s fibrils, all of which exhibited comparable efficacy (Fig. 4I).

## Discussion

The results of our study indicate that immunization with all four HET-s-derived vaccine candidates displaying conformational epitopes present on α-syn fibrils resulted in prolonged survival in immunized mice. The level of protection conferred by vaccination was dependent on the vaccine candidate used and the respective mouse model of body-first PD. We observed that the level of protection differed between the two animal models of body-first PD, with generally higher levels observed in mice challenged intragastrically. However, α-SC6 fibrils conferred better protection in intraperitoneally challenged mice. The findings indicate that the two routes of challenge, intragastric and intraperitoneal, are fundamentally distinct and influence the accessibility of pathological α-syn species to antibodies. Although the intraperitoneal challenge uses a 2-fold higher fibril dose than the intragastric route, our previously published data using the quadrivalent vaccine showed highly comparable protection (21% versus 22%), indicating that differences in administered dose alone are unlikely to explain the route-dependent effects observed here.^23^ The selected route of challenge might also result in the formation of distinct species of pathological α-syn with varying abundances of accessible antigenic epitopes.

Earlier, we reported that administration of a quadrivalent vaccine comprising equal parts of α-SC3, α-SC6, α-SC8 and α-SC9 fibrils at a dose of 100 µg resulted in a 21% increase in survival following intraperitoneal challenge and a 22% increase following intragastric challenge.^23^ In this study, we quadrupled the dose of the vaccine by using 100 µg of a single vaccine candidate. This resulted in a doubling of the level of protection provided by some of the vaccine candidates, with α-SC8 fibrils exhibiting the greatest efficacy, prolonging survival by 42% after intragastric challenge. In addition to the increased amount of a single vaccine component, it is also conceivable that removing the other HET-s-based antigens reduced potential immunological interference. In the quadrivalent formulation, immune responses directed towards any of the additional vaccine components might have competed for immune attention, whereas single-component vaccines might have allowed more focused responses towards epitopes shared across several α-syn species, including the proposed serine-rich conformational stretch.

Analysis of the relative lifespan without weight loss (Supplementary Table 3) showed that these values do not scale directly with the survival extensions reported in Table 1. After intraperitoneal challenge, α-SC6 produced the strongest survival benefit (38%) but only a moderate increase in the proportion of life spent without weight loss (72% mean-based; 65% median-based), whereas α-SC9, despite a smaller survival extension (29%), showed the highest relative preservation of weight (80% and 88%). HET-s (17%) and α-SC3 (12%) displayed relative values in the range of 66%–71%, which broadly paralleled their modest survival effects. In contrast, α-SC8, despite providing only a 6% survival extension, still showed moderately high relative weight-stable values (75% and 71%). Similar dissociations were observed after intragastric challenge. Together, these findings show that the timing of weight loss does not necessarily extend in parallel with overall survival and suggest that the constructs might differentially influence presymptomatic versus symptomatic phases rather than uniformly shifting the disease trajectory.

In addition, we cannot presently determine whether the protective effects observed here reflect sustained antibody activity throughout the course of disease or whether declining antibody titres contributed to the waning protection observed at later time points. We did not assess antibody titres at later time points post-challenge, and a reversal of seroconversion remains a plausible explanation that warrants future investigation. It also remains unknown whether administering an additional booster at these later time points could prolong protection or whether disease progression would already be too advanced for meaningful benefit. Addressing these questions will be important for evaluating whether such vaccines are best suited for prophylactic use, before or during very early peripheral α-synuclein fibrillization, or whether they also possess therapeutic potential once disease processes are underway.

It is conceivable that alterations to the dose, the timing of the boosters, the route of vaccination, and the adjuvant used might further enhance the observed health benefits. PD and other synucleinopathies primarily affect individuals >60 years of age, with incidences increasing with advancing age.^28^ A number of studies have indicated that between 30% and 50% of all cases of PD are of the body-first subtype.^27,29^ If the vaccine candidates developed and tested here prove to be protective in humans, they could delay the onset of body-first PD from an average age of 65 years to 92 years. Given that the average lifespan in the Western world is ∼80 years for both men and women, the degree of protection afforded by some of the vaccine candidates developed here would virtually eradicate body-first PD, thereby reducing the incidence of PD by almost half.^30^ It should be noted, however, that this projection assumes that vaccination is effective either prior to the initial formation of peripheral α-syn fibrils or at very early preclinical stages. Our study examines prophylactic vaccination, and whether similar levels of protection can be achieved once peripheral fibrillization has already begun remains to be determined in future work.

Interestingly, not only the four vaccine candidates displaying grafted conformational epitopes present on α-syn fibrils, but also unmodified HET-s fibrils induced significant immunity in both body-first PD models. Amino acid residues S227 in β1a in the first rung of HET-s and S263 in β3a in the second rung of HET-s form a continuous stretch of serine residues (Fig. 1F and Supplementary Fig. S8A, B), hence a conformational epitope across subunits within the HET-s fibril.^20^ Several such stretches of serine residues that form conformational epitopes across subunits are also present in the α-syn fibrils we generated to challenge TgM83^+/−^ mice (PDB ID: 8OQI), where they are formed by S42 or S87 in α-syn (Supplementary Fig. 8),^23^ which might explain how antibodies to unmodified HET-s fibrils could induce immunity to α-syn fibrils. It is also possible that HET-s fibrils induce conformation-dependent antibodies to fibrils that are independent of the amino acid sequence. Several studies have demonstrated that certain oligomeric and fibrillar antigens can induce such responses. For instance, immunization of rabbits with a homogeneous population of Aβ42 fibrils resulted in fibril-specific, conformation-dependent antibodies that recognize a generic epitope common to amyloid fibrils and fibrillar oligomers, but not random coil monomer or prefibrillar oligomers.^31^ It is of note that the fibril epitope was also identified in fibrils formed by α-syn or islet amyloid polypeptide, indicating that the epitope is a generic feature of the polypeptide backbone and not sequence specific.^31^ Furthermore, antisera of the same specificity were generated in response to immunization with islet amyloid polypeptide prefibrillar oligomer mimics and fibrils.^31^ Also, vaccination with a non-human random sequence amyloid oligomer mimic resulted in improved cognitive function and reduced plaque deposition and microhaemorrhage in an Alzheimer’s disease mouse model.^32^ In another study, a non-self peptide was covalently polymerized with glutaraldehyde until it reached a high β-sheet secondary structure content and formed species between 10 and 100 kDa that were immunogenic, stable and soluble. Inoculation of mice with this preparation resulted in the production of antibodies to antigens with β-sheet secondary structure conformation, including oligomeric conformers from Alzheimer’s, Parkinson’s and prion diseases.^33^

When we tested antibodies in plasma from immunized mice against brain homogenates from patients with synucleinopathies using competitive ELISA, we observed different results compared with when we tested the antibodies against sarkosyl-insoluble fractions prepared from brain homogenates that are enriched in α-syn fibrils.^16,17^ The antibodies in the plasma of mice immunized with HET-s and α-SC3 fibrils did not recognize DLB brain homogenates in the competitive ELISA, whereas they did recognize α-syn species present in sarkosyl-insoluble fractions prepared from brain homogenates of patients with DLB, PD and MSA. This could indicate that the fraction of recognizable α-syn fibrils is either less accessible, owing to masking, or less abundant in crude brain homogenates from DLB patients than in sarkosyl-extracts when it comes to their detection with antibodies generated to HET-s and α-SC3 fibrils. These findings also indicate that there is a variation in pathological α-syn species between DLB, PD and MSA. Together, these findings indicate that antibody recognition of synthetic fibrils does not necessarily predict *in vivo* protection; although α-SC3 elicited the strongest response to synthetic α-syn fibrils, its protective effect after intragastric challenge was not superior to that of α-SC8, α-SC9 or HET-s. This suggests that the endogenous aggregates formed in our models differ structurally from the synthetic fibrils used for immunization and seeding, and that recognition of these endogenous species is more relevant for protection.

The vaccine candidates developed and evaluated in this study were based on a small subset of conformational epitopes identified on merely two structures of synthetic α-syn fibrils (PDB IDs: 2N0A and 6H6B). Despite this limited selection, they demonstrated significant protection against the disease. We explored only a fraction of the potential conformational epitopes available for designing vaccine candidates in this research. It is probable that the inclusion of additional or different conformational epitopes could enhance immunity and disease protection. Furthermore, the recent discovery of *ex vivo* structures of α-syn fibrils extracted from the brains of individuals with synucleinopathies offers the potential to create vaccines tailored to specific synucleinopathies, which could result in more effective candidates.^16,17^

Crucially, this approach to designing conformational epitopes is of particular significance in the context of vaccine development, because it can be applied to a wide range of pathogenic protein configurations and amyloids, including amyloid β and tau, which are implicated in Alzheimer’s disease, islet amyloid polypeptide, which is relevant to type II diabetes, and other protein misfolding disorders. Furthermore, fibrillar vaccines demonstrate structural resilience, maintaining stability at room temperature over extended periods, thereby facilitating their storage, transportation and dissemination.

## Conclusion

In conclusion, our data, in conjunction with the aforementioned study,^23^ demonstrate that HET-s-derived vaccine candidates significantly delay the onset of PD-like symptoms in mouse models of PD by inducing immunity against α-syn fibrils, particularly in mouse models of body-first PD. The vaccine candidates tested here have significant clinical implications for PD and related synucleinopathies, because they could delay the onset of these diseases to later time points, when most patients die owing to health issues unrelated to PD. Therefore, these findings support the prospect of testing HET-s-based vaccine candidates in healthy individuals, and especially carriers of PD-related gene mutations, or in patients with isolated REM-sleep behaviour disorder, who eventually develop body-first PD.

## Supplementary Material

awag010_Supplementary_Data

## Data Availability

All data are available in the manuscript or the Supplementary material.
