# Supplementary material for: Vaccines mimicking conformational epitopes on α-synuclein fibrils provide immunity to Parkinson’s disease
Source: Brain. 2026 Jan 9;149(8):2689–701. doi: 10.1093/brain/awag010 (PMC13431816; doi:10.1093/brain/awag010)
Supplement: awag010_Supplementary_Data [file awag010_supplementary_data.pdf]

Vaccines mimicking conformational epitopes on α-synuclein fibrils provide immunity to Parkinson's disease

Liang Ma[1,2†], Sara Reithofer[1,2†], Verena Pesch[1,2†], José Miguel Flores-Fernandez[3¶], Aishwarya Sriraman[3], Caleb Duckering[3], Sara Amidian[3], Pelin Özdüzenciler[1,2], Laura Müller[1,2], Holger Wille[3,4*], and Gültekin Tamgüney[1,2*]

Materials and methods

Transmission electron microscopy of HET-s-derived fibrils

To verify the fibril formation of HET-s and the four vaccine candidates, negative staining transmission electron microscopy (TEM) was employed to visualize and capture images of the purified samples. Carbon-coated copper grids with a mesh size of 200 squares were discharged at 15 mA and 0.39 mBar for 1 min. Approximately 5 μL of a solution containing ~1 mg/mL was applied to the grid and allowed to absorb for 1 min. Subsequently, the grid was washed with two drops (50 μL) of ammonium acetate before staining with two drops (50 μL) of 2% filtered uranyl acetate (Electron Microscopy Sciences). Afterward, the grids were blotted dry with filter paper, stored at room temperature, and examined using a Tecnai F20 transmission electron microscope (TEM) equipped with a bottom-mounted Eagle 4k × 4k camera, operating at 200 kV.

Western blot analysis

Brain tissue was homogenized in calcium- and magnesium-free PBS (pH 7.4) supplemented with HALT protease and phosphatase inhibitor cocktail (Thermo Fisher Scientific) and benzonase (Thermo Fisher Scientific), using two 30-second cycles in a Precellys 24-Dual homogenizer (Peqlab) to yield a 20% (w/v) homogenate. Cellular debris was cleared by centrifugation at 1,000 × g for 5 minutes at 4 °C. The total protein concentration of the supernatant was measured using the Pierce BCA Protein Assay Kit (Thermo Fisher Scientific). For downstream processing, homogenates containing 1 mg of total protein were brought to 750 mM NaCl and treated with 10% (w/v) N-lauroylsarcosyl (Sigma) for 15 minutes on ice. Samples were then subjected to ultracentrifugation at 465,000 × g for 1 hour at 4 °C using a 3 mL 10% (w/v) sucrose cushion in a TLA-110 rotor (Beckman Coulter). Pellets were resuspended in 50 µL of freshly prepared TD4215 denaturing buffer containing 4% SDS, 2% β-mercaptoethanol, 192 mM glycine, 25 mM Tris, 5% (w/v) sucrose, along with HALT inhibitor cocktail and benzonase. The resuspended samples were boiled for 5 minutes and subsequently loaded onto 4–12% NuPage Bis–Tris gels (Thermo Fisher Scientific) for SDS-PAGE using a MES buffer system (Thermo Fisher Scientific). Protein bands were transferred to PVDF membranes via semidry blotting and chemically cross-linked with 0.4% (v/v) paraformaldehyde in Tris-buffered saline (Sigma) for 30 minutes at room temperature. Membranes were then blocked in TBS containing 0.05% (v/v) Tween 20 (MP Biomedical) and 5% (w/v) milk for 1 hour at room temperature, followed by overnight incubation at 4 °C with the EP1536Y antibody specific for phosphorylated α-synuclein (Abcam). After three washes in 0.05% (v/v) Tween 20 in TBS, membranes were incubated for 1 hour at room temperature with a 1:10,000 dilution of horseradish peroxidase-conjugated anti-rabbit secondary antibody (Cayman). Detection was performed using SuperSignal West Dura Extended Duration Substrate (Thermo Fisher Scientific), and chemiluminescence was imaged on a Gel Doc XR+ system (Bio-Rad).

Immunohistochemistry

For immunohistochemical analysis, formalin-fixed tissues were subjected to dehydration in a series of graded alcohol baths and ROTI-Histol (Carl Roth). Subsequently, the tissues were embedded in paraffin, sectioned into 8-µm-thick coronal slices, mounted on slides, dried overnight, and stored at 4 °C. Prior to staining, the tissue sections underwent deparaffinization

and rehydration. To retrieve antigens, the slides were incubated in citrate buffer for 10 minutes, followed by boiling in a microwave oven for an additional 10 minutes. After cooling, endogenous peroxidases were deactivated by incubating the sections in 5% hydrogen peroxide in methanol for 30 min. Next, the sections were blocked for 1 hour at room temperature in a solution containing 20% (v/v) normal goat serum, 1% (v/v) BSA, and 0.5% Triton X-100 (Sigma) in PBS. To detect α-syn phosphorylated at serine 129, the sections were exposed overnight at room temperature to a 1:1000 dilution of biotinylated pSyn#64 antibody (Wako) in a solution of 1% (v/v) normal goat serum, 1% (v/v) BSA, and 0.25% Triton X-100 in PBS. For sections stained with plasma, a 1:100 dilution of plasma was used, followed by a 1:200 dilution of biotinylated secondary anti-mouse antibody (Vector Laboratories). Following incubation, the sections were washed once with a wash buffer (0.25% (v/v) Triton X-100 in PBS) and twice with PBS, then treated with a mixture of reagents A and B from the MOM kit (Vector Laboratories) for 1 h at room temperature. After an additional wash, peroxidase activity was visualized with DAB (3-3'-diaminobenzidine) for 40 s. The reaction was halted with 3% hydrogen peroxide, followed by three washes with H_2O. Nuclei were counterstained with Meyer's hematoxylin (Carl Roth) for 6 min. Finally, the sections were washed under running tap water for 15 min, coverslipped with Aqua-Poly/Mount (Polysciences), and imaged using a Leica DM 6000 B microscope with Leica Application Suite 4.0 (Leica).

Immunofluorescence staining

Paraffin-embedded tissues were sectioned into 8-µm-thick coronal slices, mounted on slides, and subjected to deparaffinization and rehydration. Antigen retrieval was performed as detailed for immunohistochemical analysis. Following this, the samples were washed with PBS at room temperature. To mitigate tissue autofluorescence, incubation in $CuSO_4$ was carried out for 90 min at room temperature. The slides were then blocked in a solution containing 20% (v/v)

normal goat serum, 1% (v/v) BSA, and 0.5% (v/v) Triton X-100 in PBS for 1 h at room temperature. Subsequent to blocking, the sections were incubated overnight at room temperature with a primary antibody in 1% (v/v) normal goat serum, 1% (v/v) BSA, and 0.25% Triton X-100 in PBS. After washing once with a washing buffer (0.25% (v/v) Triton X-100 in PBS) and twice with PBS, the sections were stained with the appropriate Alexa Fluor 488- or Alexa Fluor 594-conjugated secondary antibodies (Thermo Fisher Scientific) in 1% (v/v) normal goat serum, 1% (v/v) BSA, and PBS for 1 hour at room temperature. Following another wash with the washing buffer and PBS, the slides were incubated with the nuclear dye DAPI (4′,6-diamidino-2-phenylindole) in PBS for 5 min. After a final wash with H_2O, the slides were coverslipped with Fluoromount medium (Sigma) and examined using an LSM 710 confocal laser-scanning microscope (Carl Zeiss). To quantify plasma staining, brainstem sections from both a mouse with pathology and a healthy animal were stained using the primary antibody of a fully vaccinated mouse at a 1:100 dilution. Staining was visualized using an LSM 710 confocal laser-scanning microscope (Carl Zeiss) at a 40× magnification.

TR-FRET

Quantification of α-syn oligomers and aggregates in mouse brain homogenates was performed using the HTRF α-synuclein aggregation detection kit (Revvity) for time resolved-fluorescence resonance energy transfer (TR-FRET) measurements. Briefly, 10-μL samples of 10% (w/v) brain homogenate diluted in 1× lysis buffer to yield 1 μg total protein were supplemented with 10 μL of a pre-mixed antibody solution containing anti-h-α-Synuclein-d2 (acceptor) and anti-h-α-Synuclein-Tb-Cryptate (donor). Samples were transferred to a HTRF 96-well low volume plate (Revvity), covered with a plate sealer, and incubated for 20 h at room temperature. Fluorescence emission was measured at 665 nm for FRET-dependent acceptor fluorescence and at 620 nm for FRET-independent donor fluorescence using a CLARIOstar microplate

reader (BMG Labtech). The ratio of both fluorescence emission values, multiplied by 10,000, is directly proportional to the amount of human α-synuclein oligomers and aggregates in each sample. The Delta F (%) value, a measure of the signal-to-background ratio, was calculated by dividing the difference between the ratio of the sample and the ratio of the negative control by the ratio of the negative control, and then multiplying the result by 100. GraphPad Prism 10 was used for statistical analysis.

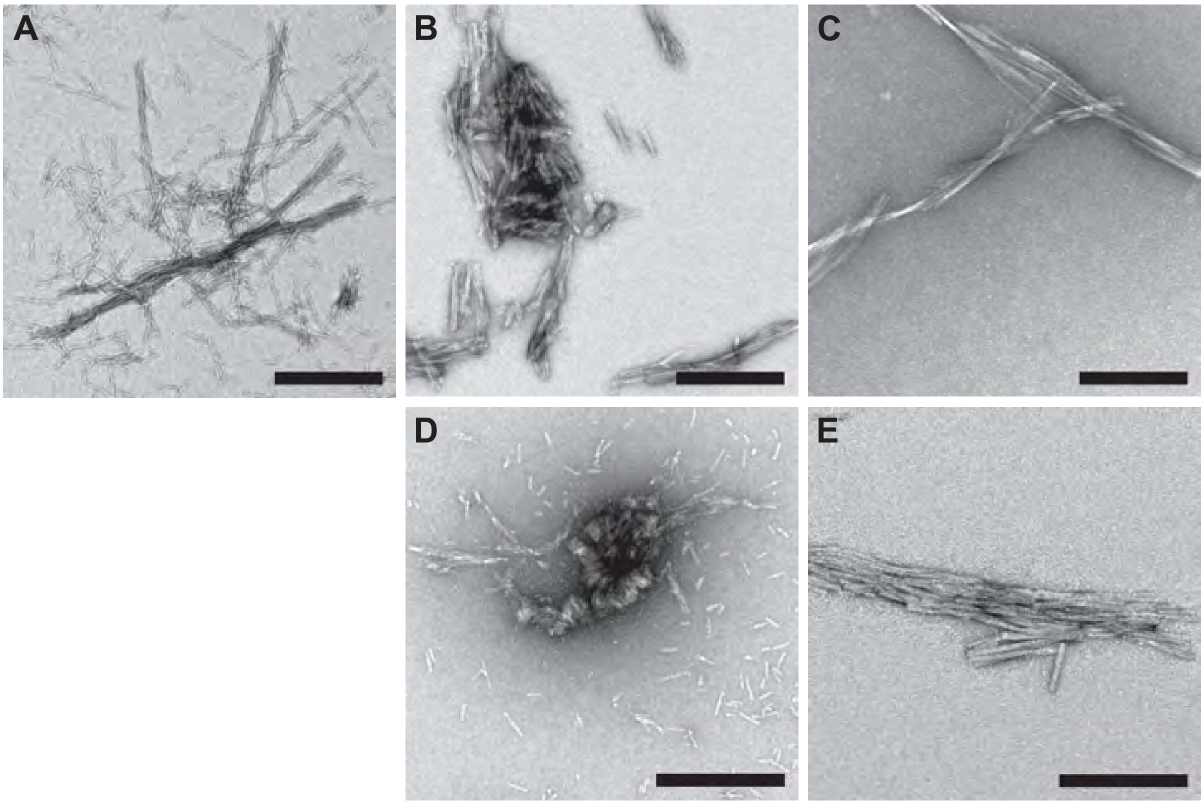

Supplementary Figure 1 HET-s and HET-s-derived vaccine candidates form fibrils. A–E Negative stain transmission electron microscopy revealed that HET-s (**A**) and all four HET-s-derived vaccine candidates, α-SC3 (**B**), α-SC6 (**C**), α-SC8 (**D**), and α-SC9 (**E**), readily formed fibrils. The scale bars represent 200 nm.

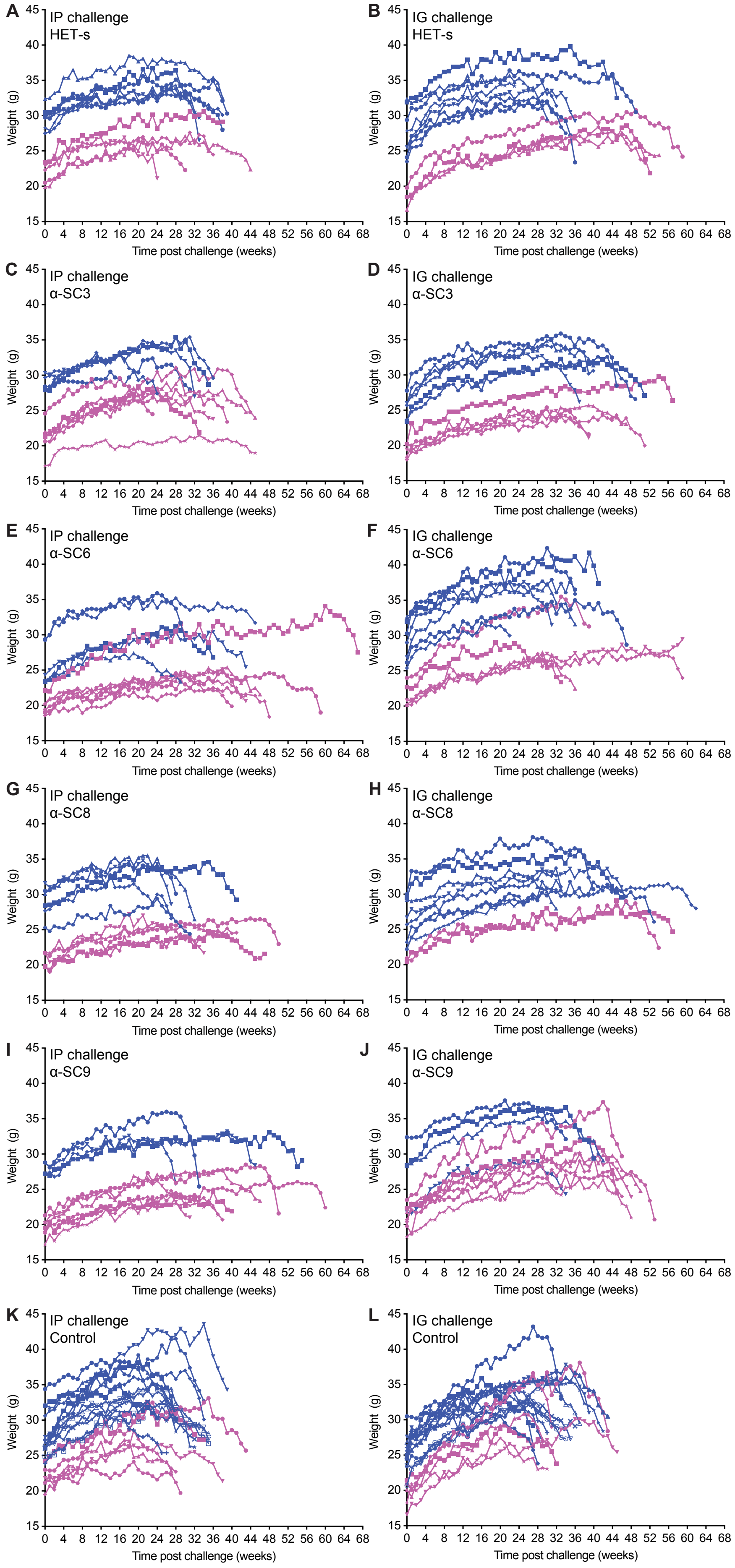
A
IP challenge
HET-s
B
IG challenge
HET-s
C
IP challenge
α-SC3
D
IG challenge
α-SC3
E
IP challenge
α-SC6
F
IG challenge
α-SC6
G
IP challenge
α-SC8
H
IG challenge
α-SC8
I
IP challenge
α-SC9
J
IG challenge
α-SC9
K
IP challenge
Control
L
IG challenge
Control
Weight (g)
15 20 25 30 35 40 45
0 4 8 12 16 20 24 28 32 36 40 44 48 52 56 60 64 68
Time post challenge (weeks)

Supplementary Figure 2 Weights of TgM83$^{+/-}$ mice challenged with α-syn fibrils. A–L Shown are the weights of intraperitoneally (**A**, **C**, **E**, **G**, **I**, and **K**) and intragastrically (**B**, **D**, **F**, **H**, **J**, and **L**) challenged mice (n = 10–12 per group) from the time of challenge to the onset of neurological disease when the mice were sacrificed. Shown are weights of mice immunized with HET-s fibrils (**A** and **B**), α-SC3 fibrils (**C** and **d**), α-SC6 fibrils (**E** and **F**), α-SC8 fibrils (**G** and **H**), α-SC9 fibrils (**I** and **J**), and of non-vaccinated control mice (**K** and **L**). The weight of male mice is indicated in blue, while that of female mice is shown in magenta.

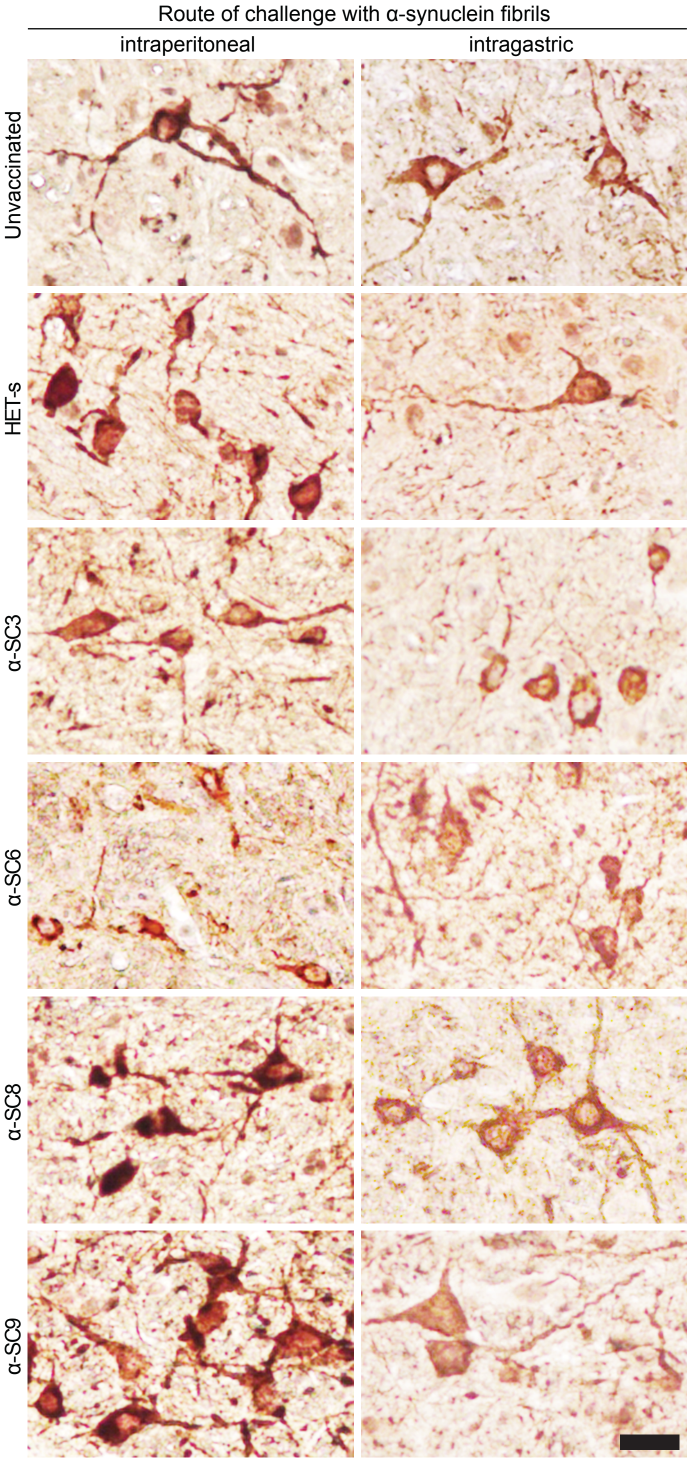

Supplementary Figure 3 Immunohistochemical analysis of brainstem sections from TgM83$^{+/–}$ mice challenged with α-syn fibrils revealed comparable levels of neuropathology in vaccinated and unvaccinated control mice at the disease end stage. Immunohistochemical staining of brainstem sections from TgM83$^{+/–}$ mice with an antibody against α-syn phosphorylated at serine 129 revealed the presence of somatic and neuritic

deposits of pathological α-syn aggregates regardless of the vaccination status or route of challenge with α-syn fibrils. Shown are images for mice vaccinated with HET-s, α-SC3, α-SC6, α-SC8, α-SC9 fibrils, and for unvaccinated control animals (top to bottom) challenged intraperitoneally (left) or intragastrically (right). The scale bar represents 50 μm and is applicable to all panels.

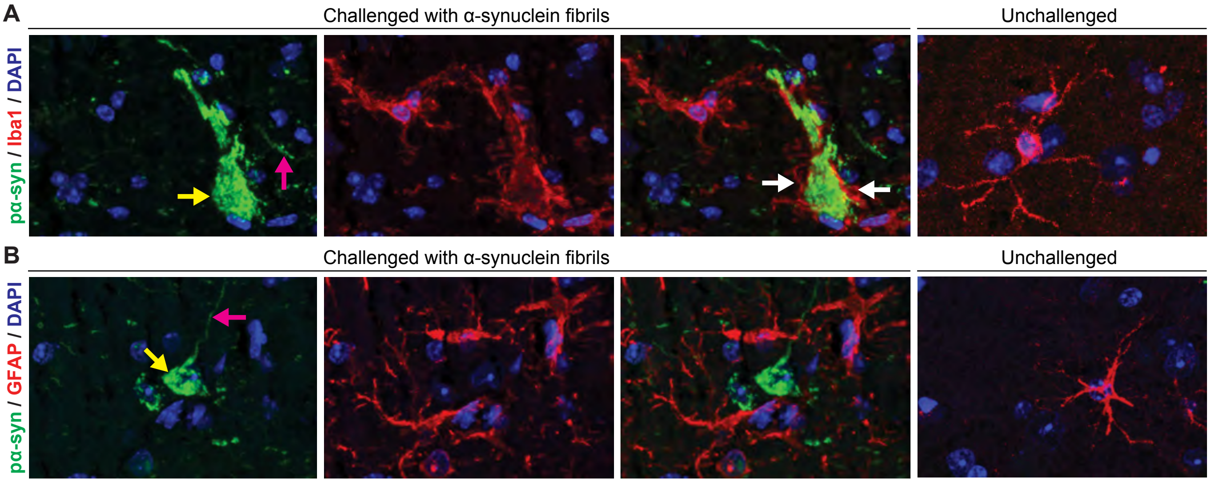

Supplementary Figure 4 Immunofluorescence analysis of the CNS of sick TgM83$^{+/-}$ mice reveals evidence of neuroinflammation. (**A**) Brainstem sections from sick TgM83$^{+/-}$ mice stained with antibodies against α-syn phosphorylated at serine 129 (green) and ionized calcium-binding adaptor molecule 1 (Iba1, red) demonstrated the presence of somatic (yellow arrow) and neuritic (magenta arrow) α-syn deposits in neurons surrounded by activated, amoeboid microglia (white arrows), indicative of microgliosis, regardless of the vaccination status or route of challenge with α-syn fibrils. The merged images of stained brainstem sections from age-matched unchallenged mice (right) demonstrated the absence of pathological changes and the presence of predominantly quiescent, ramified microglia. Nuclei were stained blue with DAPI. (**B**) Immunofluorescence analysis of brainstem sections from sick TgM83$^{+/-}$ mice with antibodies against glial fibrillary acidic protein (GFAP, red) and α-syn phosphorylated at serine 129 (green) revealed the presence of somatic (yellow arrow) and neuritic (magenta arrow) α-syn deposits in neurons, as well as an accumulation of astrocytes indicative of astrogliosis, regardless of the vaccination status or route of challenge with α-syn fibrils. Merged images of stained brainstem sections from age-matched unchallenged mice (right) demonstrated the absence of pathology and a minimal astrocyte population. Nuclei were stained blue with DAPI.

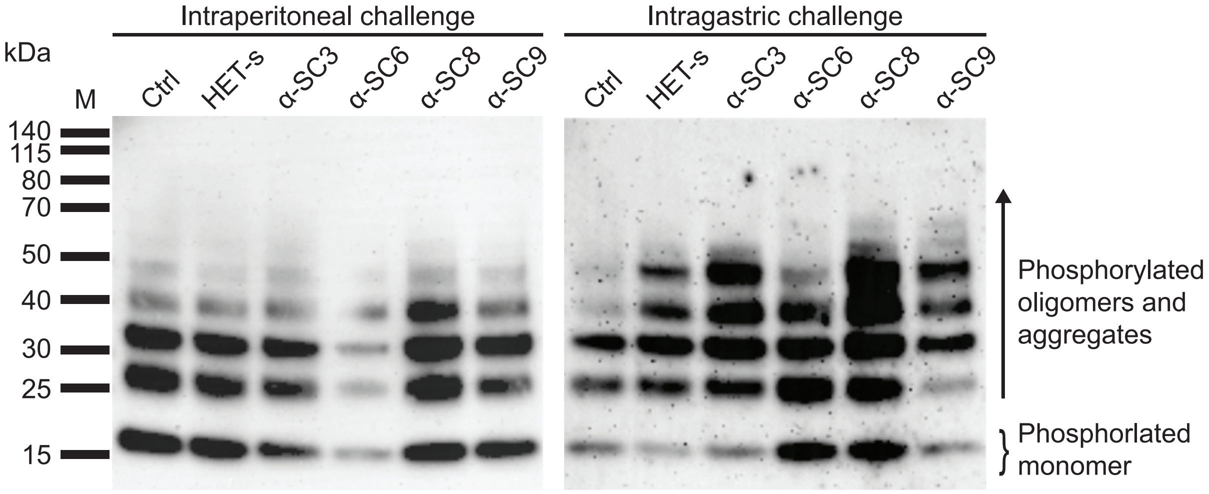

Supplementary Figure 5 Brain homogenates from sick TgM83$^{+/-}$ mice contain high molecular weight species of α-syn phosphorylated at serine 129. Western blot analysis of sarkosyl-insoluble brain homogenate fractions from unvaccinated mice (ctrl) and mice vaccinated with HET-s, α-SC3, α-SC6, α-SC8, or α-SC9 fibrils revealed the presence of diverse molecular weight species of α-syn phosphorylated at serine 129, ranging from monomers to larger assemblies, in both intraperitoneally (left) and intragastrically (right) challenged mice. The molecular weight marker (M) is shown in kilodaltons (kDa).

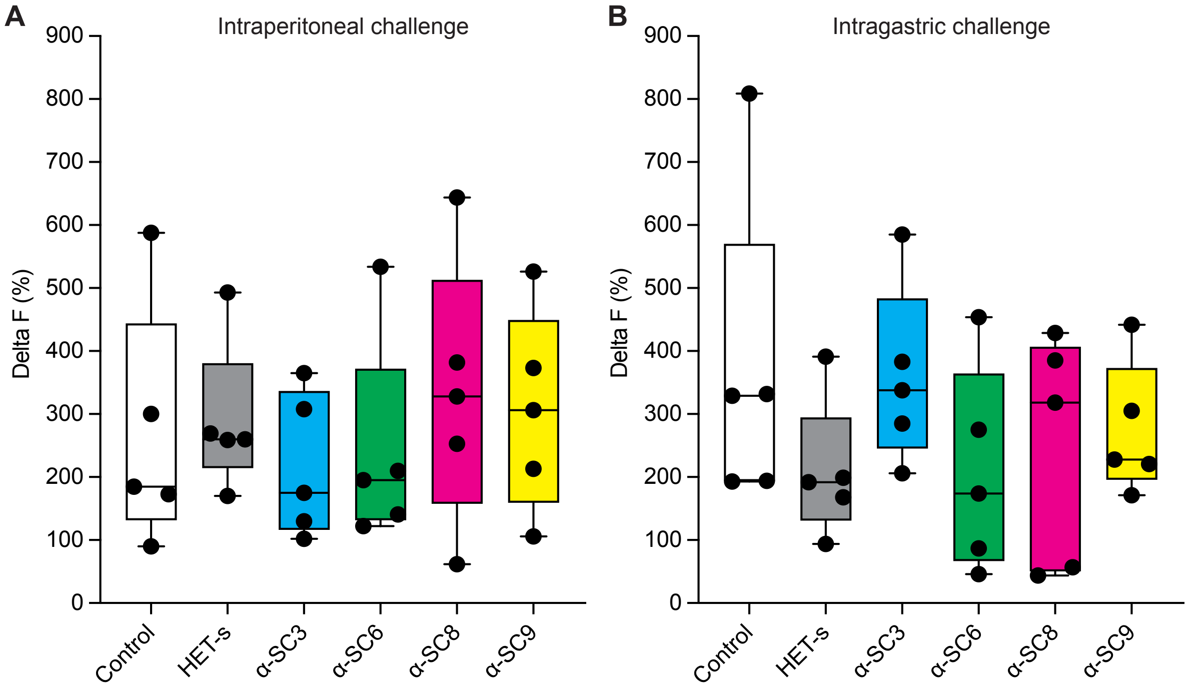

Supplementary Figure 6 Quantitative analysis with TR-FRET demonstrated comparable levels of α-syn aggregates in brain homogenates from TgM83$^{+/-}$ mice when diseased. (**A** and **B)** At the time of death, the amount of α-syn aggregates in brain homogenates from intraperitoneally (**A**) and intragastrically (**B**) challenged TgM83$^{+/-}$ mice was not significantly different between unvaccinated controls and those vaccinated with HET-s, α-SC3, α-SC6, α-SC8, or α-SC9 fibrils. The significance was assessed using one-way ANOVA, followed by Tukey's multiple comparison test. The boxes indicate the 25th to 75th percentiles. The central lines within the boxes represent the median values. Whiskers indicate the minimum and maximum values.

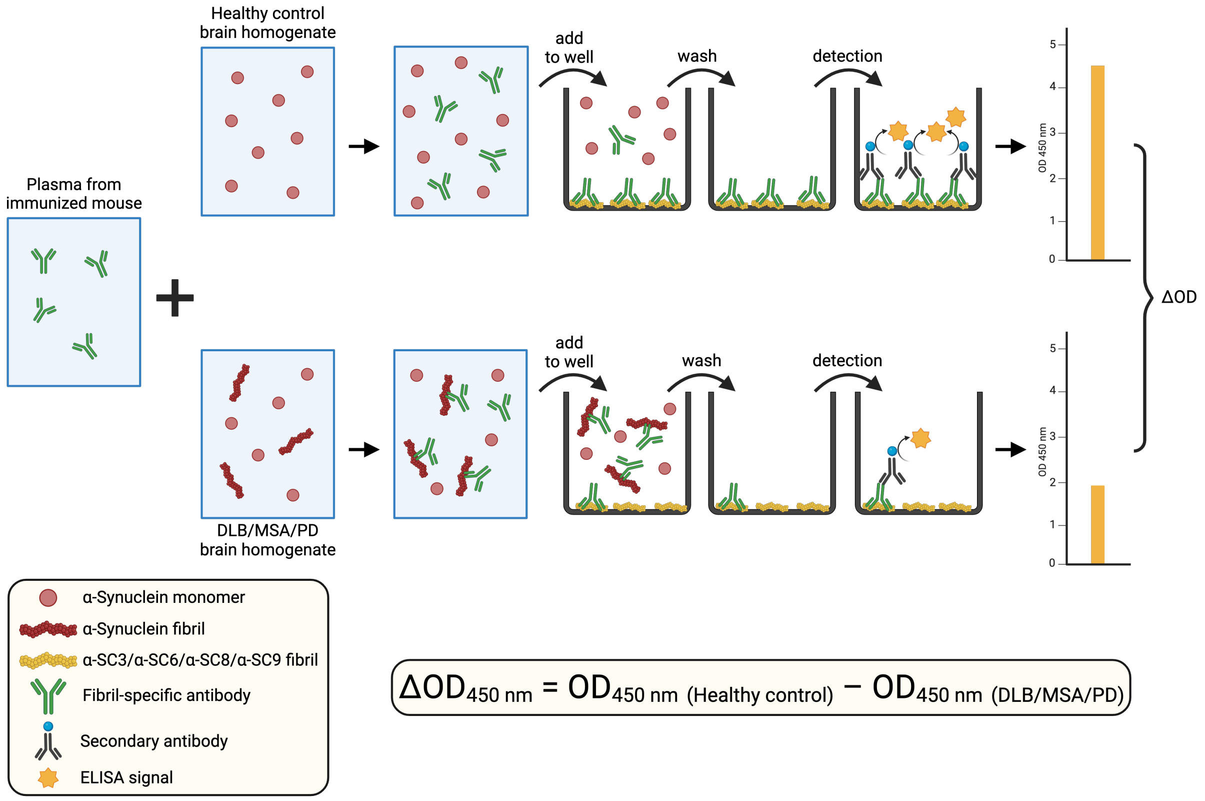

Supplementary Figure 7 Principle of the competitive ELISA. For the competitive ELISA, blood plasma from an immunized mouse was mixed with brain homogenates from non-neurological (healthy) controls (HC, top row) and patients with DLB, MSA, or PD (bottom row). Since brain homogenates from non-neurological (healthy) controls contain only monomeric α-syn and no α-syn fibrils, the majority of antibodies in the plasma of immunized mice are free to bind to wells precoated with HET-s, α-SC3, α-SC6, α-SC8, or α-SC9 fibrils, resulting in a relatively high ELISA signal [$OD_{450\ nm\ (Healthy\ control)}$]. In contrast, brain homogenates from patients with synucleinopathies, such as dementia with Lewy bodies (DLB), multiple system atrophy (MSA), and Parkinson's disease (PD), contain pathological α-syn fibrils that are bound by antibodies in the plasma of immunized mice. This results in a reduction in the number of antibodies that are free to bind to wells precoated with HET-s, α-SC3, α-SC6, α-SC8, or α-SC9 fibrils, which in turn leads to a relatively low ELISA signal [$OD_{450\ nm\ (DLB/MSA/PD)}$]. The competitive ELISA signal $\Delta OD_{450\ nm}$ is defined as the difference between the ELISA signal obtained with the non-neurological (healthy) control brain homogenate [OD_{450}

$_{nm\ (Healthy\ control)}$] and the ELISA signal obtained with the pathological brain homogenate [$OD_{450\ nm\ (DLB/MSA/PD)}$]. A $\Delta OD_{450\ nm}$ value greater than zero indicates the presence of antibodies in the plasma of fully immunized mice that recognize pathological α-syn fibrils.

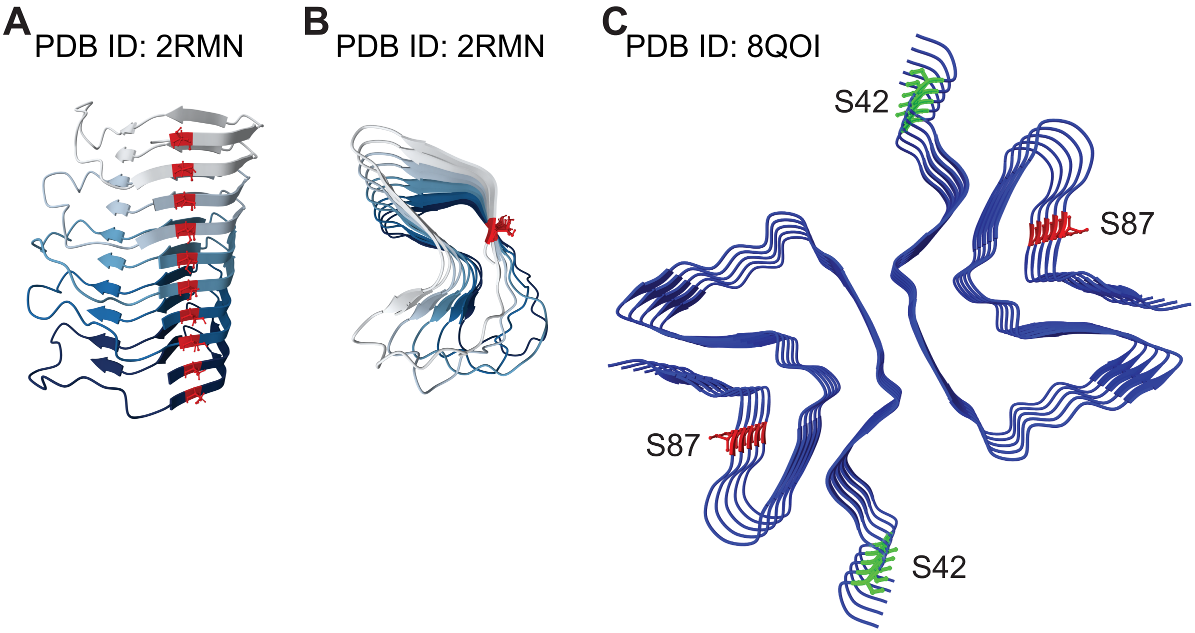

Supplementary Figure 8 Conformational epitopes composed solely of serine residues are present on HET-s and α-syn fibrils. (**A** and **B**) Amino acid residues S227 in β1a in the first rung of HET-s and S263 in β3a in the second rung of HET-s form a continuous stretch of serine residues and thus a conformational epitope across subunits within the HET-s fibril, as observed from the side (**A**) or the top (**B**) of the fibril. (**C**) Several stretches of serine residues that form conformational epitopes across subunits are also present in the α-syn fibrils generated to challenge TgM83$^{+/-}$ mice (PDB ID: 8OQI). These epitopes are formed by S42 or S87 in α-syn, which may explain how antibodies to unmodified HET-s fibrils could induce immunity to α-syn fibrils.

Supplementary Table 1 Human brain tissues used for competitive ELISA

Sample ID	Diagnosis	Sex	Age	Brain region	Braak LB stage
HC1	HC	m	72	Medulla oblongata	0
HC2	HC	f	80	Medulla oblongata	0
HC3	HC	f	55	Substantia nigra	0
HC4	HC	f	78	Putamen	0
HC5	HC	m	69	Medulla oblongata	0
DLB1	DLB	f	86	Caudate with putamen	5
DLB2	DLB	m	78	Caudate with putamen accumbens	6
DLB3	DLB	m	72	Caudate with putamen accumbens	6
MSA1	MSA	f	61	Cerebellum	–
MSA2	MSA	m	57	Cerebellum	–
MSA3	MSA	m	73	Cerebellum	–
PD1	PD	m	72	Caudate with putamen accumbens	5
PD2	PD	m	76	Caudate with putamen accumbens	6
PD3	PD	m	82	Caudate with putamen accumbens	6

HC healthy control, *DLB* dementia with Lewy bodies, *MSA* multiple system atrophy, *PD* Parkinson's disease, *m* male, *f* female, *LB* Lewy body

Supplementary Table 2 Human brain tissues used for TR-FRET

Sample ID	Diagnosis	Sex	Age	Brain region	Braak LB stage
DLB	DLB	m	78	Substantia nigra	6
PD	PD	m	84	Caudate with putamen accumbens	5
MSA	MSA	f	71	Cerebellum	–

DLB dementia with Lewy bodies, *PD* Parkinson's disease, *MSA* multiple system atrophy, *m* male, *f* female, *LB* Lewy body

Supplementary Table 3 Relative life span without weight loss

Route of challenge	Unvaccinated control[23]	HET-s	α-SC3	α-SC6	α-SC8	α-SC9
**Intraperitoneal**						
Rel. life span without weight loss	68	69	68	72	75	80
Rel. life span without weight loss	69	71	66	65	71	88
**Intragastric**						
Rel. life span without weight loss	76	77	73	82	75	77
Rel. life span without weight loss	76	72	73	84	71	81

[a] Values represent the mean period without weight loss relative to the mean incubation period.

[b] Values represent the median period without weight loss relative to the median incubation period.
